# Impact of Dry and Rainy Seasons on the Chemical Profile and Antioxidant Activity of *Lippia alba* Essential Oil

**DOI:** 10.3390/molecules31061035

**Published:** 2026-03-20

**Authors:** Rodrigo Dias Alves, João Pedro Bauman Quieregati, Julia Samara Pereira de Souza, Maria Helena Brandão-Silva, Ariana Pereira da Silva, Katia Castanho Scortecci, Jacqueline do Carmo Barreto, Hugo Alexandre Oliveira Rocha

**Affiliations:** 1 Graduate Program in Biochemistry and Molecular Biology, Center of Biosciences, Federal University of Rio Grande do Norte—UFRN, Natal 59078-970, RN, Brazil; rodrigofarmaufcg@gmail.com (R.D.A.); jpquieregati@gmail.com (J.P.B.Q.); arianapereirauf@gmail.com (A.P.d.S.); kacscort@yahoo.com (K.C.S.); 2 Graduate Program in Health Science, Center of Health Science, Federal University of Rio Grande do Norte—UFRN, Natal 59078-970, RN, Brazil; juliasamara16@gmail.com; 3 Natural Polymer Biotechnology Laboratory (BIOPOL), Department of Biochemistry, Center of Biosciences, Federal University of Rio Grande do Norte—UFRN, Natal 59078-970, RN, Brazil; helena.brandao.129@ufrn.edu.br; 4 Laboratory of Plant Transformation and Microscopy Analysis (LTPAM), Department of Cell Biology and Genetics, Federal University of Rio Grande do Norte—UFRN, Natal 59078-970, RN, Brazil; 5 Academic Unit of Biology and Chemistry (UABQ), Federal University of Campina Grande—UFCG, Cuité 58175-000, PB, Brazil; jackie_barreto74@yahoo.com.br

**Keywords:** seasonal variation, cytoprotection, phytochemical profile, antiproliferative activity, metabolomic variation, reactive oxygen species

## Abstract

Seasonal environmental conditions can modulate the chemical composition and biological activity of essential oils from medicinal plants. This study investigated the phytochemical profile, antioxidant potential, cytotoxic activity, and cytoprotective effects of *Lippia alba* essential oils collected during dry and rainy seasons. Gas chromatography analysis revealed that all samples preserved a citral chemotype. Principal Component Analysis (PCA) confirmed citral as the primary discriminant metabolite, while quantitative seasonal variations were mainly associated with minor oxygenated monoterpenes, particularly geraniol, carvone, and nerolidol. The essential oil obtained during the rainy season (A5T–RS) exhibited significantly higher antioxidant activity, as determined by 2,2-diphenyl-1-picrylhydrazyl (DPPH), reducing power, total antioxidant capacity, and hydrogen peroxide scavenging assays. Intracellular reactive oxygen species (ROS) evaluation using the 2′,7′-dichlorodihydrofluorescein diacetate (DCFH-DA) method demonstrated that both oils reduced oxidative stress in murine fibroblasts—L929, with enhanced cytoprotective effects observed for A5T–RS. Cytotoxicity assays against non-tumor (murine fibroblast-NIH/3T3, L929, Chinese hamster ovary—CHO-K1) and tumor (human cervical carcinoma—HeLa, and human hepatocellular carcinoma—HepG2) cell lines revealed selective antiproliferative activity, with tumor cells displaying greater sensitivity, particularly to the rainy-season oil. These results demonstrate that seasonal metabolomic modulation enhances the biological performance of *L. alba* essential oil without altering its chemotypic identity, highlighting the importance of environmental factors in the development of bioactive plant-derived products.

## 1. Introduction

The term medicinal plant refers to any plant species capable of producing, in significant concentrations, chemical or biological substances that can be applied directly or indirectly as therapeutic agents. In recent decades, scientific and commercial interest in medicinal plants has increased substantially, driven by the growing demand for natural and sustainable products, partly due to concerns regarding adverse effects and long-term toxicity associated with synthetic drugs [1,2]. Preclinical and clinical studies have demonstrated that several species traditionally used in folk medicine are rich sources of natural compounds, such as flavonoids, alkaloids, triterpenes, tannins, and phenolic compounds, which exhibit a wide range of pharmacological activities [3,4]. Recent advancements in metabolomics and biotechnological tools have further accelerated the identification of these bioactive scaffolds, reinforcing their role in modern drug development [5].

Among the plant species of important ethnobotanical and pharmacological relevance, *Lippia alba* (Mill.) N.E. Brown, belonging to the family Verbenaceae, stands out. This family comprises approximately 32 to 35 genera and more than 1000 species, with a predominantly pantropical distribution [6]. In Brazil, Verbenaceae is represented by a significant diversity, with many species presenting recognized economic and medicinal potential [7]. Species of the genus *Lippia* exhibit broad ecological adaptability, occurring from humid tropical regions to semi-arid environments. This adaptability contributes to pronounced metabolic plasticity, resulting in distinct chemotypes that vary according to environmental stressors and geographical location [8].

Ethnobotanical studies indicate that *L. alba* is among of the most widely used medicinal plants in Brazil, particularly in the treatment of gastrointestinal disorders [9], inflammation conditions [10], and central nervous system-related disorders, such as anxiety and insomnia [11]. In traditional medicine, its leaves are prepared through various methods, including infusions and tinctures, and are administered for both internal and external therapeutic purposes [12].

Many of these therapeutic properties have been associated with the chemical composition of the essential oil of *L. alba*, which is recognized as a rich source of mono- and sesquiterpenes, including compounds such as citral (neral and geranial), linalool, carvone, limonene, citronellal, geraniol, β-caryophyllene, p-cymene, γ-terpinene, α-pinene, and thymol [13]. In addition to volatile constituents, the species also contains non-volatile metabolites such as flavonoids, iridoids, alkaloids, triterpenes, and saponins, which collectively contribute to its diverse pharmacological profile [14,15].

The chemical composition of *L. alba* essential oils exhibits pronounced variability, supporting the existence of multiple chemotypes within the species. Consequently, several authors have proposed the classification of *L. alba* based on the major constituents of its essential oils [16]. In Brazil, at least three main chemotypes have been reported, namely citral, carvone, and linalool types [17]. According to Matos [18], each chemotype is associated with specific biological activities. For example, chemotypes characterized by myrcene–citral or limonene–citral have been associated with tranquilizing, analgesic, anxiolytic, sedative, and antispasmodic effects, whereas the limonene–carvone chemotype has been primary linked to mucolytic action and the treatment of gastrointestinal disorders.

In addition to the intrinsic chemical variability observed among chemotypes the biosynthesis and accumulation of secondary metabolites in *L. alba* are strongly influenced by environmental conditions. Factors such as temperature, rainfall, humidity, solar radiation, and seasonality are recognized as key drivers of qualitative and quantitative variations in essential oil composition [19]. In this context, seasonal contrasts between dry and rainy periods may impose distinct physiological stresses on plants, potentially modulating metabolic pathways involved in terpenoid biosynthesis. However, despite Brazil’s marked climatic heterogeneity, most studies addressing chemical variability in plants have focused on commercially exploited species from temperate regions [20], and investigations evaluating the seasonal influence of dry and rainy periods on native Brazilian species remain limited. This gap underscores the importance of studies aimed at understanding how seasonal dynamics affect the chemical profile of *L. alba* essential oils, particularly under contrasting hydric conditions.

In this context, a detailed evaluation of seasonal effects on the chemical profile of *L. alba* essential oil is necessary to better understand the relationship between environmental conditions and secondary metabolism in this species. Although the occurrence of different chemotypes has been well documented, information on how contrasting hydric regimes, such as dry and rainy seasons, modulate the qualitative and quantitative composition of the essential oil remains fragmented, particularly for native populations from tropical regions. Therefore, the present study investigates the influence of dry and rainy seasons on the chemical composition and biological activity of *L. alba* essential oil, using chromatographic analyses combined with biochemical and cell-based assays. By correlating seasonal variability with changes in chemical profile and bioactivity, this study provides relevant insights for the standardization of plant raw materials, the definition of optimal harvesting periods, and the rational pharmacological exploitation of this species.

## 2. Results and Discussion

### 2.1. Essential Oil Extraction and Yield from the Leaves of Lippia alba Mill.

Table 1 summarizes the essential oil yields obtained from nine consecutive collections. Yields ranged from 0.070% to 0.628% (*w*/*w*). Samples collected during the dry season exhibited consistently higher yields (0.279–0.628%; mean = 0.427%) compared to those obtained during the rainy season (0.070–0.273%; mean = 0.171%).

These values fall within the typical range reported for aromatic plants (~0.2%) [21] and are consistent with previous reports for *L. alba* (0.1–1.2%) [13,22]. The variation observed among the samples reflects the significant influence of seasonal and temporal factors on essential oil production. In particular, the data suggest that quantitative differences are closely associated with the collection period, likely driven by changes in precipitation and solar radiation that affect glandular trichome productivity [23].

Higher yields during the dry season likely reflect physiological adjustments under water limitation. According to the growth–defense trade-off framework, plants subjected to abiotic stress may preferentially allocate carbon to secondary metabolism rather than biomass production [24], a response previously described for *L. alba* under water stress conditions [15].

Conversely, the lower yields observed during the rainy season can be partially attributed to increased tissue hydration at harvest. Because yield was calculated based on fresh mass, higher water content may result in a dilution effect of essential oil concentration within vegetative tissues [25,26].

The variations observed based on the collection time further indicate the potential influence of circadian rhythms on the production and accumulation of volatile compounds. The trend toward higher yields in morning collections may be associated with the daily dynamics of metabolites biosynthesis and storage. In *L. alba*, this process is regulated by abiotic signaling, such as light intensity and temperature, which modulates the activity of key enzymes during the early hours of the day [15,27,28].

Taken together, essential oil productivity reflects the combined effects of seasonality and diurnal variation rather than a single isolated factor. These findings reinforce the importance of defining optimal harvest conditions to improve raw material standardization and maximize extraction efficiency [29]. 

### 2.2. Seasonal Variation in the Chemical Composition of L. alba Leaf Essential Oil

The chemical composition of the essential oil obtained from *L. alba* leaves collected at different times of the day and during different seasons is summarized in Table 2. The essential oil showed a predominance of oxygenated monoterpenes, with citral identified as the major constituent in all analyzed samples, ranging from 42.32% to 80.69%.

In addition to citral, other relevant compounds were identified at varying proportions, including D-limonene, geraniol, and carvone, which contributed significantly to the chemical profile of the essential oil. D-limonene contents ranged from 2.58% to 8.02%, while geraniol varied from 3.85% to 16.65%, with higher concentrations generally observed in samples collected during the dry season. Carvone was detected in all samples, with sample A8M–DS exhibiting the highest content of this compound (11.18%).

The predominance of oxygenated monoterpenes, particularly citral (up to 80.69%), identifies the specimens from Cuité, Paraiba State (Brazil), as belonging to the citral chemotype [30], which is well adapted to the high solar radiation and thermal stress characteristic of the Brazilian semi-arid [31].

The increase in geraniol during the dry season, together with the elevated carvone content (11.18%) observed in sample A8M–DS, suggests a metabolic redirection in response to environmental pressures. As reported by Shan et al. [32], high solar radiation typical of tropical semi-arid climates acts as an environmental signal that upregulates monoterpene biosynthesis, particularly favoring the accumulation of oxygenated monoterpenes in plant tissues as protective compounds under intense light stress. Moreover, Sharma et al. [33] showed that these fluctuations in major constituents are not random, but rather reflect adaptive modulation of enzymatic pathways, especially those regulating the competition between geraniol and limonene-derived metabolites, thereby optimizing chemical defense during periods of increased abiotic stress through enhanced flux via the MEP pathway.

Minor constituents such as β-myrcene, 1-octen-3-ol, linalool, geranyl acetate, and nerolidol were detected in variable proportions, contributing to quantitative diversity among samples.

Rainy-season oils generally displayed lower citral percentages and greater compositional diversity, including citronellol and higher nerolidol levels, indicating quantitative seasonal modulation while maintaining chemotypic identity.

Collectively, these data demonstrate that *L. alba* exhibits metabolic plasticity driven by seasonal and diurnal factors, with stable chemotype but significant quantitative shifts in secondary metabolites.

### 2.3. Principal Component Analysis (PCA)

Principal component analysis (PCA) was applied as an exploratory multivariate approach to facilitate the interpretation of the chemical variability among the *L. alba* essential oil samples. PCA reduces dataset complexity by condensing multiple chemical variables into a limited number of new variables, known as principal components, which capture the main sources of variation among the samples.

In the present study, the first two principal components (PC1 and PC2) explained 33.3% and 25.0% of the total variance, respectively, accounting for 58.3% of the overall chemical variability. For complex natural matrices such as essential oils, this proportion of explained variance is considered adequate and allows a reliable interpretation of chemical patterns using the PC1 × PC2 biplot.

**Figure 1:**
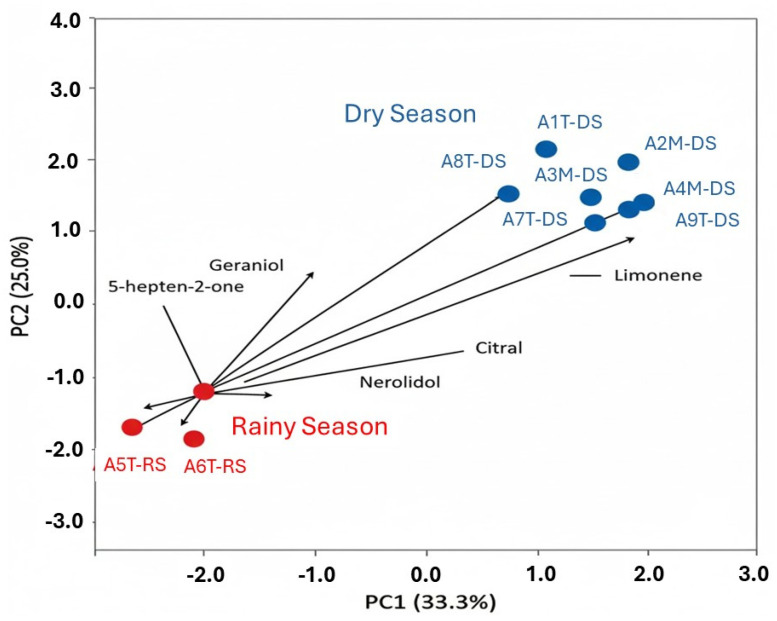
Principal Component Analysis (PCA) biplot of *Lippia alba* essential oil chemical composition. The first two components (PC1 and PC2) account for 58.3% of the total cumulative variance. Samples are grouped by seasonal collection: Dry Season (blue circles) and Rainy Season (red circles). Vectors represent the load of major chemical constituents. PC1 (33.3%) is primarily influenced by citral content, while PC2 (25.0%) reflects seasonal modulation driven by minor constituents such as geraniol, nerolidol, and 5-hepten-2-one. Rainy-season samples (A5T-EC and A6M-EC) are displaced along PC2, indicating higher chemical diversity and enrichment of secondary metabolites compared to the dry-season cluster.

The PCA biplot (Figure 1) simultaneously illustrates the distribution of the samples (scores) and the contribution of the individual chemical constituents (loadings). In this representation, samples located in proximity exhibit similar chemical compositions, whereas the direction and length of the vectors indicate the relative contribution of each compound to sample differentiation.

A well-defined chemical pattern was observed, with citral displaying the highest loading on PC1 (loading = 0.874), as evidenced by its long vector predominantly aligned along the PC1 axis. This high loading magnitude indicates a strong positive contribution of citral to the variance captured by PC1. This result indicates that citral is the principal variable driving chemical differentiation and the primary chemical identity among all analyzed samples. From a phytochemical perspective, the predominance of a single compound along the main axis of variation is a widely accepted criterion for chemotype definition. Accordingly, the PCA results provide strong statistical support for classifying all analyzed samples as belonging to the citral chemotype.

Seasonal effects were primarily associated with the second principal component (PC2), which was influenced by secondary constituents such as geraniol, nerolidol, and 5-hepten-2-one. While samples collected during the rainy season (A5T-EC and A6M-EC) are positioned farther from the citral vector and closer to these secondary compounds, they remain within the same chemical domain as the dry season samples.

Despite variation in collection time (morning or afternoon) and seasonal conditions (dry and rainy), all samples clustered within the same chemical region characterized by a high contribution of citral. This indicates that these environmental factors induced quantitative shifts in compound concentrations, such as the higher chemical diversity observed in the rainy season, rather than qualitative changes in chemical composition. In conclusion, the PCA confirms that *L. alba* essential oils collected in Cuité–Paraíba State (Brazil) exhibit a single and stable citral chemotype, where seasonal and diurnal factors influence but do not alter the dominant chemical identity of the oil.

Taken together, the PCA confirms that *L. alba* essential oils collected in Cuité–Paraíba State (Brazil) exhibit a single and stable citral chemotype. Seasonal and diurnal factors influence but do not alter the dominant chemical identity of the oil.

### 2.4. In Vitro Antioxidant Activity of L. alba Leaf Essential Oil

Considering the chemical profiles obtained, samples A5T–RS (rainy season) and A1T–DS (dry season) were selected for the evaluation of antioxidant activity in vitro and in vivo. The selection of sample A1T–DS was justified by its high citral content (80.69%), together with the presence of oxygenated monoterpenes such as geraniol and carvone, which are widely reported for their antioxidant properties and capacity to scavenge reactive oxygen species [34,35]. This sample represents a more concentrated and chemically defined profile, characteristic of the citral chemotype, allowing the assessment of the antioxidant potential of an essential oil dominated by a major bioactive compound.

In contrast, sample A5T–RS was selected due to its lower citral content (42.32%) and higher chemical diversity, including mono- and sesquiterpene derivatives, enabling investigation of potential synergistic effects among minor constituents on antioxidant activity. Comparison of samples collected during the dry and rainy seasons therefore provides insight into the influence of seasonal variation on the biological activity of *L. alba* essential oil and contributes to a better understanding of the relationship between chemical composition and biological effects.

Catechin was included as a reference antioxidant control to validate assay responsiveness and to provide a comparative benchmark for radical scavenging and redox-related measurements. Its inclusion allows contextualization of the relative antioxidant potency of the essential oil samples without altering the primary comparative focus between seasonal extracts.

The antioxidant activities of the *L. alba* essential oils are presented in Table 3. Antioxidant responses were significantly influenced by both seasonality and concentration (*p* < 0.05). Among the evaluated samples, A5T–RS extract at 1.0 mg/mL consistently exhibited the highest antioxidant performance across all assays. In the DPPH radical scavenging assay, most treatments showed baseline activity levels of approximately 23%, whereas A5T–RS at the highest concentration displayed a twofold increase (48.33 ± 4.73%). A similar trend was observed in the reducing power, hydrogen peroxide scavenging, and total antioxidant capacity (TAC) assays, in which A5T–RS at 1.0 mg/mL significantly outperformed the dry-season counterpart (A1T–DS). These results indicate that antioxidant efficiency is strongly modulated by seasonal chemical variation rather than solely by concentration effects, consistent with previous reports showing that essential oils bioactivity depends on quantitative shits in chemical composition [36].

As expected, catechin exhibited high antioxidant activity across the evaluated assays, confirming the methodological sensitivity and reliability of the experimental system. However, the comparative interpretation of seasonal essential oil samples remains independent of the reference compound, as the primary objective of this study was to elucidate how seasonal modulation within a stable chemotype affects intrinsic antioxidant performance.

The A5T–RS sample exhibited a pronounced increase in antioxidant activity at 1.0 mg/mL, whereas A1T–DS maintained comparable activity levels across both tested concentrations. This pattern suggests that, although citral predominates in the chemical profile of all analyzed samples, the enhanced radical scavenging activity observed at higher concentrations is more closed associated with the contribution of minor oxygenated monoterpenes. Previous studies have shown that compounds such as geraniol [37] and carvone [38] possess significant antioxidant potential, and may modulate or even exceed the activity of major constituents within essential oils. In addition, the presence of nerolidol in rainy-season samples may further reinforce this effect, as sesquiterpenoids are known to stabilize reactive species and enhance the overall antioxidant capacity of terpene-rich matrices [39]. This interpretation is consistent with classical studies demonstrating that oxygenated monoterpenes, including geraniol and carvone, exhibit pronounced radical scavenging properties [40].

The interpretation of antioxidant results is strongly supported by principal component analysis (PCA), which identified citral as the main discriminant compound defining the chemical profile of all analyzed samples. The PCA biplot showed that citral exhibited the highest loading along PC1, which accounted for the largest proportion of chemical variance among samples. Importantly, all extracts clustered within the same chemical domain, confirming the presence of a single citral chemotype regardless of seasonal or diurnal collection conditions. Samples collected during the rainy season displayed slight displacement along PC2, reflecting variations in the relative abundance of minor oxygenated constituents rather than the emergence of distinct chemotypes. This statistical pattern corroborates the biochemical interpretation that seasonal environmental factors promote quantitative metabolic modulation without altering the fundamental phytochemical identity of the essential oil.

The pronounced superiority of A5T–RS at 1.0 mg/mL, particularly in the DPPH assay, suggests the occurrence of an entourage effect, in which biological activity arises from synergistic interactions among multiple constituents rather than from a single dominant compound. Recent studies have emphasized that the biological effects of essential oils often result from cooperative interactions among several metabolites rather than from the isolated action of the major component [41,42]. The displacement of rainy-season samples along PC2 in the PCA supports this hypothesis, as this component was associated with increased contributions from geraniol, nerolidol, and other oxygenated terpenes. Therefore, the superior antioxidant performance observed for A5T–RS reinforces the interpretation that seasonal modulation of minor constituents enhances bioactivity while preserving the overall chemotypic identity of *L. alba* essential oil.

A comparative analysis among the antioxidant assays further highlights the involvement of distinct redox mechanisms underlying extract activity. The DPPH assay evaluates hydrogen atom transfer capacity and is considered a sterically demanding radical model system [43]. The selective effectiveness of A5T–RS at higher concentration suggests that rainy-season metabolites possess enhanced hydrogen-donating properties. In contrast, the reducing power assay assesses electron-transfer mechanisms by measuring the ability of antioxidants to reduce ferric ions (Fe^3+^) into ferrous ions (Fe^2+^), representing an alternative antioxidant defense pathway [44]. Similarly, the total antioxidant capacity (TAC) assay, conducted under acidic and elevated temperature conditions, estimates the global electron-donating potential of complex extracts under chemically stressful environments. Together, these assays demonstrate that *L. alba* essential oils exhibit a multi-target antioxidant capacity, acting through complementary hydrogen transfer and electron donation pathways, in accordance with recommendations for comprehensive antioxidant evaluation [45].

Seasonal variation appears to play a key role in modulating the secondary metabolism of *L. alba*. Samples collected during rainy-season exhibited a relative enrichment of oxygenated monoterpenes, indicating metabolic adjustment associated with adaptation to environmental stress. Climatic variables such as humidity, temperature fluctuations, and increased microbial or herbivore pressure have been shown to influence terpene biosynthesis and promote the accumulation of oxygenated derivatives in aromatic plants [46]. This metabolomic plasticity directly affects phytochemical complexity and may enhance the pharmacological potential of essential oils, as reflected by the improved antioxidant performance of the rainy-season extract.

The superior activity observed in the hydrogen peroxide scavenging assay is particularly relevant from a biological perspective, as this test evaluates the capacity of extracts to neutralize reactive oxygen species before the formation of highly reactive hydroxyl radicals via Fenton-type reactions [47]. Hydrogen peroxide plays a central role in oxidative stress signaling and cellular damage pathways; therefore, its neutralization represents a critical antioxidant defense mechanism. When considered alongside the total antioxidant capacity (TAC) assay, which reflects global electron-donating ability under chemically stressful conditions, the results indicate that the rainy-season extract possesses a broader and more versatile antioxidant defense profile. These findings further support the notion that seasonal modulation of minor constituents enhances biological functionality while preserving the fundamental citral chemotype of *L. alba* essential oil. Hydrogen peroxide plays a central role in oxidative stress signaling and cellular damage pathways; therefore, its neutralization represents a critical antioxidant defense mechanism. When considered alongside the total antioxidant capacity (TAC) assay, which reflects global electron-donating ability under chemically stressful conditions, the results indicate that the rainy-season extract possesses a broader and more versatile antioxidant defense profile. These findings further support the notion that seasonal modulation of minor constituents enhances biological functionality while preserving the fundamental citral chemotype of *L. alba* essential oil.

Taken together, the PCA and antioxidant data suggest that although citral defines the chemotype and dominates the chemical profile, the enhanced antioxidant activity observed for the rainy-season oil at higher concentration is primary associated with synergistic interactions among minor oxygenated constituents. Compounds such as geraniol, nerolidol, and carvone are known contributors to antioxidant mechanisms, and their increased relative abundance in rainy-season samples may potentiate radical scavenging activity once a critical concentration threshold is reached.

Therefore, the antioxidant response observed in this study appears to be driven by quantitative compositional shifts within a stable chemotype rather than by the emergence of distinct chemical types. This finding highlights the importance of considering both major and minor constituents, as well as their interactions, when correlating phytochemical profiles with biological activities.

#### Integrated Chemometric Analysis: PCA and Chemical Diversity–Activity Relationship

Principal Component Analysis (PCA) was initially performed to explore compositional differences between A1T–DS and A5T–RS essential oils. The multivariate model clearly separated the samples, indicating distinct chemical fingerprints associated with each sampling condition. The loading plot demonstrated that A1T–DS was strongly associated with citral predominance, whereas A5T–RS clustered with a broader distribution of oxygenated monoterpenes and minor constituents.

While PCA efficiently highlighted compositional differentiation, it does not directly quantify chemical complexity. Therefore, to complement the multivariate analysis, the Shannon diversity index (H′) was calculated based on the relative abundance of identified constituents.

A1T–DS exhibited a low diversity index (H′ = 0.82), reflecting a chemically concentrated profile dominated by citral (80.69%). In contrast, A5T–RS presented a markedly higher diversity index (H′ = 1.94), indicating a more evenly distributed phytochemical composition, with reduced citral content (42.32%) and increased proportions of geraniol, D-limonene, β-myrcene, nerolidol, and other oxygenated monoterpenes.

This shift from a dominance-driven to a diversity-driven chemical profile suggests a transition from a single-compound-centered system to a multicomponent matrix. Such increased compositional diversity may enhance antioxidant performance through additive or synergistic interactions among bioactive molecules. Oxygenated monoterpenes, particularly alcohol-containing structures such as geraniol and nerolidol, are known to contribute to redox modulation via hydrogen-donating capacity and stabilization of reactive species.

Importantly, due to the experimental design, single pooled extraction per condition, correlation or regression analyses between individual compounds and biological activity were not statistically feasible. Consequently, the present data do not allow quantitative attribution of antioxidant activity to specific constituents or estimation of relative contribution percentages. However, the integration of PCA clustering patterns with chemical diversity metrics provides mechanistic plausibility linking compositional complexity to functional antioxidant differences observed between A1T–DS and A5T–RS.

Taken together, the combined chemometric approach indicates that antioxidant activity does not depend exclusively on citral content, but rather on the synergistic balance and diversity of oxygenated constituents within the essential oil matrix. These findings underscore the role of chemical complexity as a key determinant of bioactivity in phytochemical systems.

In addition to diversity metrics, a comparative assessment of major constituents versus antioxidant performance suggests that bioactivity cannot be solely attributed to the most abundant compound. Although A1T–DS was strongly dominated by citral (80.69%), this chemical concentration did not proportionally translate into superior antioxidant performance when compared to A5T–RS. Conversely, A5T–RS, despite presenting lower citral content (42.32%), exhibited a more chemically distributed profile enriched in oxygenated monoterpenes such as geraniol and nerolidol. This observation indicates that antioxidant capacity may depend not only on the quantitative predominance of a single aldehydic compound but also on the qualitative contribution of multiple redox-active constituents. Such findings reinforce the concept that phytochemical matrices often display emergent bioactivity resulting from compositional balance rather than dominance alone.

### 2.5. Seasonal Metabolomic Modulation and Cytotoxic Activity

The cytotoxic potential of *L. alba* essential oils obtained from dry-season (A1T–DS) and rainy-season (A5T–RS) collections was evaluated against non-tumor and tumor cell lines, and the IC_50_ values are summarized in Table 4. Overall, both samples exhibited cell line-dependent cytotoxicity, with markedly enhanced antiproliferative effects toward tumor cells, particularly for the rainy-season oil. These findings suggest that seasonal environmental conditions influence not only chemical composition but also the biological performance of the essential oils.

Among non-tumor fibroblast lineages, A1T–DS displayed low cytotoxicity, with IC_50_ values exceeding 100 µg/mL for NIH/3T3 cells and 120 µg/mL for L929 cells, indicating a favorable safety profile. In contrast, A5T–RS showed moderately increased activity, with IC_50_ values of 85.21 µg/mL (NIH/3T3) and 95.32 µg/mL (L929). CHO-K1 cells were comparatively more sensitive to both oils, particularly A5T–RS (IC_50_ = 65.23 µg/mL). This potentially highlights lineage-specific variations regarding the cytotoxic pathways triggered by terpene-rich matrices.

In tumor cell lines, both essential oils demonstrated substantially greater antiproliferative effects. HeLa cells were the most susceptible, especially to A5T–RS, which exhibited the lowest IC_50_ value observed in this study (35.36 µg/mL). A similar pattern was observed for HepG2 cells, where A5T–RS (IC_50_ = 45.52 µg/mL) showed significantly higher potency than A1T–DS (IC_50_ = 70.21 µg/mL). This selective cytotoxicity toward tumor-derived cells, combined with lower toxicity to normal fibroblasts, indicates that seasonal variation may enhance therapeutic selectivity without increasing general cytotoxic risk.

Chemometric analysis provides strong support for this biological behavior. PCA demonstrated that all samples clustered within a single citral chemotype, with citral acting as the principal discriminant variable along PC1. However, rainy-season samples were displaced along PC2, reflecting a relative enrichment of oxygenated monoterpenes and sesquiterpenoids, including geraniol, carvone, and nerolidol. This pattern indicates that seasonal environmental pressures do not induce chemotypic shifts but instead promote seasonal metabolomic modulation, characterized by quantitative reorganization of secondary metabolite abundance.

Such metabolomic plasticity is commonly associated with plant adaptive responses to environmental stimuli, including variations in humidity, temperature, pathogen pressure, and herbivory. Increased biosynthesis of oxygenated derivatives during the rainy season may enhance plant chemical defense mechanisms, which, in turn, can amplify the pharmacological potential of the resulting extracts. The present results suggest that the same metabolite ensemble responsible for enhanced antioxidant performance also contributes to increased cellular bioactivity, with selective effects depending on cellular metabolism.

Citral is well documented as an inducer of apoptosis in cancer cells through mitochondrial dysfunction, ROS generation, and modulation of MAPK and p53-dependent pathways [48]. However, increasing evidence suggests that minor oxygenated constituents can significantly potentiate these effects. Geraniol and carvone have been shown to exert antiproliferative activity by induces apoptosis and cell cycle arrest, modulates multiple molecular targets, including p53 and STAT3, activates caspases, and modulates inflammation via transcriptional regulation [38,49], while nerolidol contributes to membrane permeabilization and oxidative imbalance in tumor cells [39]. The co-occurrence of these compounds in A5T–RS supports a synergistic or “entourage effect”, enhancing bioactivity beyond what would be expected from citral alone [40,41].

The determination of the Selectivity Index (SI) is a critical parameter in pharmacological prospecting, as it quantifies the balance between a compound’s therapeutic efficacy and its safety. Although some IC_50_ values for non-tumor cells were not reached at the highest tested concentrations (particularly for the A1T–DS sample), these results are highly positive from a toxicological standpoint. They demonstrate that the essential oil’s baseline toxicity is low, establishing a minimum safety margin that exceeds the concentration required to inhibit tumor cell growth.

The Selectivity Index further reinforces the relevance of seasonal metabolomic modulation. A5T–RS exhibited an SI of 2.43 when comparing NIH/3T3 fibroblasts to HeLa cells, exceeding the threshold generally proposed for selective cytotoxicity in natural product research [49]. In contrast, A1T–DS showed lower selectivity and higher IC_50_ values across all lineages, consistent with its reduced abundance of minor oxygenated metabolites identified by PCA. These findings demonstrate that quantitative seasonal changes in secondary metabolism can directly influence pharmacological selectivity while preserving chemotypic identity.

Taken together, these findings demonstrate that seasonal variation modulates the relative composition of secondary metabolites (metabolomic modulation) in *L. alba* essential oil without altering its chemotypic identity. The enhanced cytotoxic activity and selectivity of the rainy-season oil are closely associated with quantitative shifts in minor constituents captured by PCA, highlighting the importance of chemometric approaches for linking chemical variability to biological performance. In Addition, this approach underscores the importance of integrating chemometric tools with pharmacological assays to better understand how environmental factors influence the therapeutic potential of aromatic plants.

### 2.6. Cellular Protection Against Hydrogen Peroxide-Induced Oxidative Damage

The cytoprotective effects of *L. alba* essential oils against oxidative stress were evaluated using L929 fibroblast cells exposed to hydrogen peroxide, and the results are summarized in Table 5. Exposure to H_2_O_2_ alone significantly reduced cell viability, confirming the effectiveness of the oxidative stress model. Cells treated exclusively with hydrogen peroxide exhibited a marked decrease in viability, reaching approximately 47% compared to untreated control cells.

Pretreatment with both essential oils significantly attenuated oxidative damage in a concentration-dependent manner. The dry-season oil (A1T–DS) promoted moderate cytoprotection, increasing cell viability from 62% at 5 µg/mL to 78% at 25 µg/mL. In contrast, the rainy-season oil (A5T–RS) exhibited a more pronounced protective effect across all concentrations, restoring cell viability to approximately 68%, 78%, and 88% at 5, 10, and 25 µg/mL, respectively. Statistical analysis followed by Tukey’s post hoc test demonstrated that both oils significantly improved cell survival compared to the H_2_O_2_-treated group (*p* < 0.05). Moreover, A5T–RS at the highest concentration exhibited significantly greater cytoprotective activity than A1T–DS, indicating enhanced biological efficacy of the rainy-season oil.

Importantly, treatment with either essential oil in the absence of oxidative stress did not significantly alter cell viability compared to untreated control cells, suggesting low intrinsic cytotoxicity under the tested conditions. This observation supports the safety profile of these oils within the evaluated concentration range and reinforces their potential application as protective agents against oxidative injury.

Overall, the results demonstrate that the essential oils of *L. alba* exhibit protective effects against oxidative stress-induced cellular damage, with the rainy-season oil displaying enhanced cytoprotective performance. This behavior is likely associated with seasonal variations in secondary metabolite composition, particularly the higher relative abundance of oxygenated monoterpenes detected in the rainy-season sample.

### 2.7. Evaluation of Oxidative Status via DCFH-DA Fluorescence

The protective effects of *L. alba* essential oils against H_2_O_2_-induced oxidative stress in L929 fibroblasts are presented in Table 6. Exposure to H_2_O_2_ (300 µM) significantly increased ROS generation to 195.0%, compared to the negative control (100%). Pre-treatment with both essential oils (A1T–DS and A5T–RS) resulted in a dose-dependent reduction in ROS levels. Notably, the A5T–RS sample at 25 mg/mL showed the highest protective activity, reducing ROS levels to 115.2%, which was significantly more effective than the A1T–DS sample at the same concentration (148.3%). These results suggest that the antioxidant potential varies according to the seasonal collection of essential oils.

The intracellular ROS assay demonstrated that hydrogen peroxide exposure significantly increased oxidative stress in L929 fibroblasts, confirming the effectiveness of the oxidative injury model. Pretreatment with *L. alba* essential oils attenuated ROS accumulation in a concentration-dependent manner, although the magnitude of protection differed according to seasonal origin. The rainy-season oil (A5T–RS) produced a significantly stronger reduction in intracellular ROS compared to the dry-season sample (A1T–DS), indicating enhanced cytoprotective capacity. Importantly, neither essential oil induced ROS overproduction when applied alone, suggesting low intrinsic pro-oxidant activity and supporting their biological safety under the tested conditions.

The differential intracellular antioxidant performance observed between seasonal samples appears to be directly associated with quantitative variations in secondary metabolite composition. The A1T–DS sample exhibited moderate cytoprotective activity consistent with its chemical profile dominated by citral. Citral is known to exert antioxidant and cytoprotective effects through direct radical scavenging and modulation of mitochondrial redox balance [50]. However, studies indicate that citral alone often displays limited efficiency in stabilizing intracellular oxidative cascades when compared to complex terpene mixtures.

In contrast, the A5T–RS oil exhibited markedly superior ROS suppression and cellular protection, which can be attributed to the higher abundance of oxygenated monoterpenes and sesquiterpenes such as geraniol, carvone, and nerolidol. These metabolites contribute to redox regulation through multiple complementary mechanisms. Geraniol has been shown to enhance antioxidant enzyme activity and modulate membrane stability through hydrogen atom transfer reactions [51]. Carvone acts as an efficient electron donor and has demonstrated the ability to modulate lipid peroxidation and mitochondrial oxidative stress [38]. Nerolidol, in turn, has been reported to increase membrane permeability and induce controlled oxidative imbalance in malignant cells while contributing to cytoprotective responses in normal tissues [39]. The coexistence of these metabolites supports the occurrence of a synergistic or “entourage effect,” in which cooperative interactions among constituents enhance overall biological activity [40,41].

These intracellular antioxidant findings are consistent with the results obtained in chemical antioxidant assays. The rainy-season oil exhibited superior radical scavenging capacity in the DPPH assay, suggesting greater efficiency in neutralizing highly stable radicals. Additionally, the reducing power and total antioxidant capacity assays demonstrated that A5T–RS possesses a multi-target antioxidant profile, indicating that this sample can act through both hydrogen atom transfer and electron transfer mechanisms. The strong correlation between chemical antioxidant performance and cellular ROS modulation reinforces the relevance of multi-mechanistic redox activity in determining cytoprotective potential.

Chemometric analysis provides further insight into the relationship between chemical variability and biological activity. PCA demonstrated that all essential oil samples belong to a single citral chemotype, with citral acting as the main discriminant variable along PC1. However, rainy-season samples exhibited displacement along the PC2 axis, reflecting enrichment in minor oxygenated metabolites. This pattern indicates that the observed biological effects are not associated with chemotypic variation, but rather with seasonal metabolomic modulation. This process is characterized by quantitative shifts in metabolite abundance driven by environmental factors. Similar adaptive metabolic plasticity has been widely reported in aromatic plants exposed to seasonal fluctuations in humidity, temperature, and pathogen pressure [20,52].

Interestingly, the same metabolite ensemble responsible for enhanced intracellular antioxidant performance also appears to influence selective cytotoxicity toward tumor cells. Cytotoxicity assays demonstrated that A5T–RS exhibited significantly stronger antiproliferative effects against HeLa and HepG2 tumor cell lines while maintaining lower toxicity toward normal fibroblasts. This selective cytotoxic behavior is consistent with the dual redox-modulating capacity of terpene-rich essential oils. Moderate ROS modulation in normal cells tends to promote cytoprotection and maintenance of redox homeostasis, whereas tumor cells, which typically present elevated basal oxidative stress, are more susceptible to redox imbalance and mitochondrial dysfunction [53,54].

Citral has been widely reported to induce apoptosis in tumor cells through mitochondrial depolarization, activation of caspase cascades, and modulation of MAPK signaling pathway [55]. However, increasing evidence indicates that oxygenated monoterpenes can significantly potentiate these effects. Geraniol has been shown to induce cell cycle arrest and inhibit tumor cell proliferation through modulation of oxidative stress signaling [55]. Carvone has demonstrated antiproliferative activity through mitochondrial ROS modulation and lipid membrane destabilization [38]. Nerolidol contributes to enhanced tumor cell permeability and apoptosis induction through oxidative stress amplification [39]. The synergistic combination of these metabolites likely explains the lower IC_50_ values and higher Selectivity Index observed for A5T–RS.

The Selectivity Index values obtained in this study further support the pharmacological relevance of seasonal metabolomic modulation. A5T–RS exhibited an SI greater than 2.0 when comparing fibroblast and tumor cell responses, a threshold commonly used to identify promising anticancer candidates in natural product research [49]. These findings demonstrate that quantitative seasonal variation in secondary metabolism can simultaneously enhance antioxidant defense and tumor-selective cytotoxicity while maintaining cellular safety in non-malignant models.

Collectively, the results highlight seasonal metabolomic modulation as a key determinant of the biological performance of *L. alba* essential oils. Environmental seasonality does not alter the fundamental chemotypic identity of the oil but instead reshapes metabolite abundance, generating distinct redox-modulating and cytotoxic profiles. The integration of chemometric tools with cellular and biochemical assays provides a comprehensive understanding of how environmental variability influences the therapeutic potential of aromatic plants and reinforces the importance of minor metabolites in determining biological functionality.

Together, these findings indicate that the antioxidant capacity demonstrated in chemical assays translates functionally into intracellular redox stabilization, thereby constituting a central mechanism underlying the cytoprotective effects observed in fibroblast cells.

### 2.8. Study Limitations

Although the chemical and biological patterns observed were consistent throughout the study, some limitations warrant consideration. First, while nine oil samples were analyzed, only two corresponded to the rainy season. This distribution reflects the typical climatic reality of the semi-arid region of Northeastern Brazil (Cuité, Paraíba State), where precipitation is highly seasonal, irregular, and concentrated in short hydrological windows. Thus, the rainy-season samples accurately characterize the local environmental regime during the study period rather than a sampling constraint. Nevertheless, we acknowledge that broader interannual evaluations would further strengthen the robustness of these seasonal comparisons.

Regarding the biological assays, we selected two representative afternoon samples (dry and rainy seasons) based on their pronounced chemical contrast, as supported by the PCA clustering pattern. The afternoon samples exhibited the most significant seasonal divergence, particularly in the abundance of minor oxygenated constituents associated with redox-related bioactivity. While we acknowledge that circadian rhythms may influence metabolite levels, our multivariate analysis indicated that seasonal modulation was the primary driver of chemical variation. Future research incorporating a full factorial biological evaluation of all time points and multi-year collections would be valuable to further clarify the interplay between circadian dynamics and interannual climatic variability in *L. alba* bioactivity.

## 3. Materials and Methods

### 3.1. Plant Material Collection

*L. alba* leaves (approximated one year old plants) were collected along the margins of Lagoa de Cuité, located in the municipality of Cuité, Paraíba State, Brazil (geographical coordinates: 6°28′54″ S, 36°08′59″ W). Sampling was carried out over a nine-month period, including collections performed both in the morning and in the afternoon. In addition, plant material was collected during both the dry and rainy seasons to evaluate possible seasonal variations in essential oil yield and composition, with March and April representing the rainy period and May to November corresponding to the non-rainy season. Collection was authorized by the Brazilian National System of Management of Genetic Heritage and Associated Traditional Knowledge (SISGEN registration no. ADD87FB).

**Figure 2:**
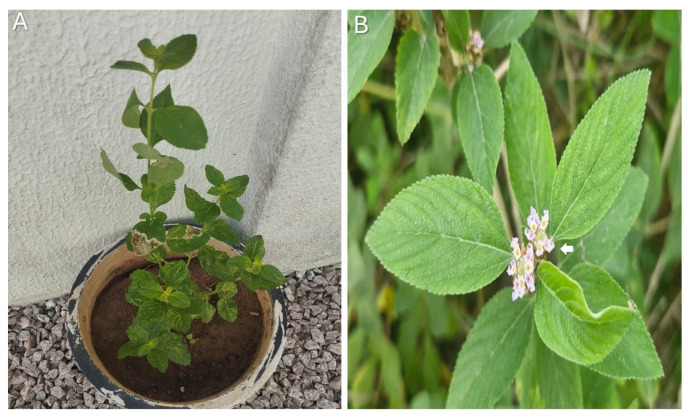
Morphological characteristics of *L. alba* (Mill.) N.E. Brown. (**A**) General view of the plant. (**B**) Close-up view highlighting the inflorescences; the arrow indicates the flowers.

*L. alba* (Mill.) N.E. Brown (Verbenaceae) is a perennial aromatic shrub, reaching approximately 1.0–2.0 m in height, with a highly branched habit and quadrangular, pubescent to glabrescent stems that become woody at the base. Leaves are opposite, simple, ovate to elliptic, with serrate to crenate margins and a pubescent surface rich in glandular trichomes responsible for the characteristic aroma. The inflorescences are axillary or terminal, forming dense globose to ovoid capitula with small, sessile flowers, whose corolla ranges from white to pale lilac. The species produces a small schizocarpic fruit and exhibits marked intraspecific variability, particularly regarding essential oil composition. Representative morphological characteristics of *L. alba* are shown in Figure 2.

### 3.2. Essential Oil Extraction

Essential oils were obtained by hydrodistillation using a modified Clevenger-type apparatus. Fresh leaves were collected from 15 individual *L. alba* plants and pooled to minimize intra-population variability within each seasonal sampling. The collected material was triturated using a domestic blender (BMP900, 1000W, Britania, Linhares, ES, Brazil) until homogeneous fragmentation was achieved.

The mass of fresh triturated leaves used for each seasonal extraction ranged from 39.432 g to 47.604 g. The plant material was transferred to a 1 L round-bottom flask containing 500 mL of distilled water and subjected to hydrodistillation for 2 h from the onset of boiling under atmospheric pressure using an electric heating mantle (Cole-Parmer™ HM-200 Series Flask/Funnel Heating Mantle with Controller; Cole-Parmer, Vernon Hills, IL, USA) with temperature control.

Volatile constituents were co-distilled with water vapor, condensed through a vertical condenser, and collected in the graduated arm of the Clevenger apparatus. After completion of distillation, the essential oil layer was carefully separated from the aqueous phase (hydrolate) using a Pasteur pipette.

The essential oil was dried over anhydrous sodium sulfate to remove residual moisture, filtered, and transferred to 2 mL amber glass vials. Samples were hermetically sealed and stored at −5 °C, protected from light, until GC–MS analysis. Essential oil yield (% *w*/*w*) was calculated relative to the fresh plant material mass and is presented in Table 1.

For each seasonal condition, a single pooled extraction was performed, and thus the chemical profile represents the integrated metabolomic composition of the sampled population rather than technical extraction replicates.

### 3.3. Gas Chromatography–Mass Spectrometry (GC–MS) Analysis

Gas chromatography analyses were performed using a Hewlett–Packard HP 5890 gas chromatograph coupled to a Hewlett–Packard HP 5971 mass-selective detector (Hewlett–Packard, Palo Alto, CA, USA). Samples (1 μL) were injected into the GC system, and the separation of volatile constituents was achieved using a fused-silica capillary column coated with dimethylpolysiloxane (DB-1; 30 m × 0.25 mm i.d.). Helium was employed as the carrier gas at a constant flow rate of 1.0 mL/min.

The oven temperature program was set as follows: initial temperature of 35 °C, increased from 35 to 180 °C at a rate of 4 °C/min, followed by a ramp from 180 to 250 °C at 10 °C/min. The injector temperature was maintained at 250 °C, and the transfer line temperature was set at 280 °C.

Mass spectra were acquired in electron impact (EI) ionization mode at 70 eV, with the ion source temperature set to 200 °C and a filament current of 34.6 μA. Owing to the high reproducibility of EI mass spectra at 70 eV, compound identification was performed by comparison of the obtained spectra with those available in commercial mass spectral libraries.

Individual components were identified based on mass spectral matching with two MS databases, as well as by comparison of their calculated Kovats retention indices with literature values. Additional confirmation was obtained through visual comparison with previously reported data.

Representative GC–MS total ion chromatograms (TIC) and mass spectra used for compound identification are provided in the Supplementary Material (Figure S1).

### 3.4. In Vitro Evaluation of the Antioxidant Activity of the Samples

To obtain a comprehensive antioxidant profile, the samples were evaluated using multiple in vitro assays based on distinct and complementary antioxidant mechanisms. The Total Antioxidant Capacity (TAC) assay estimates the overall electron-donating potential of thermally stable compounds under strong oxidative conditions; the reducing power assay reflects electron transfer capacity and redox potential; the copper chelation assay evaluates preventive antioxidant activity through transition metal sequestration; and the hydrogen peroxide scavenging assay assesses the ability to neutralize a biologically relevant reactive oxygen species. In addition, the DPPH radical scavenging assay, commonly used to evaluate hydrogen- or electron-donating capacity toward stable free radicals, was performed.

#### 3.4.1. Total Antioxidant Capacity Assay (TAC)

The Total Antioxidant Capacity (TAC) assay is widely used to determine the overall reducing potential of a sample by measuring its capacity to donate electrons under strongly oxidative conditions. The reaction is conducted under non-physiological experimental parameters, such as high temperature, acidic medium, and the presence of a strong oxidizing system, which allows the estimation of the maximal reducing power of thermostable antioxidant molecules present in the sample. In this context, the assay reflects the cumulative electron-donating ability of antioxidant constituents with broad reducing activity.

The determination of TAC was performed following a protocol adapted from Costa et al. [56]. In this assay, electron transfer from antioxidant compounds promotes the reduction of molybdenum from the hexavalent oxidation state (Mo^6+^) to the pentavalent form (Mo^5+^) in an acidic environment, which serves as a general indicator of the reducing capacity of the tested material. This process leads to the formation of a green phosphate/Mo^5+^ complex that can be quantified spectrophotometrically. For the analysis, sample aliquots were combined with the reagent solution composed of 0.6 M sulfuric acid, 28 mM sodium phosphate, and 4 mM ammonium molybdate, followed by thermal incubation at 100 °C for 90 min. After cooling the reaction mixture to room temperature, the absorbance was recorded at 695 nm using a spectrophotometer. Ascorbic acid was employed as the reference compound, and the antioxidant activity was expressed as ascorbic acid equivalents (AAE).

#### 3.4.2. Reducing Power Assay

The reducing power assay evaluates the capacity of antioxidant compounds to act as electron donors, promoting the conversion of ferric ions (Fe^3+^) into their ferrous form (Fe^2+^). This reaction serves as an indicator of the redox properties of the tested sample and is frequently linked to antioxidant mechanisms capable of interrupting radical chain reactions by preventing the propagation of reactive species.

The reducing activity of the samples was determined according to the procedure reported by Presa et al. [57]. For the essay, sample solutions (1 mg/mL) were mixed with 0.2 M phosphate buffer (pH 6.6) and 1% (*w*/*v*) potassium ferricyanide, followed by incubation at 50 °C for 20 min to allow the reduction reaction to occur. The reaction was subsequently terminated by the addition of 10% (*w*/*v*) trichloroacetic acid (TCA), and 0.1% (*w*/*v*) ferric chloride was then added to enable color development. Ascorbic acid was used as the reference antioxidant compound. Absorbance was recorded spectrophotometrically at 700 nm. The results were expressed as the percentage of reducing power relative to the activity of ascorbic acid, considering the response obtained at a concentration of 0.1 mg/mL as the reference value. The percentage of reducing power was calculated according to the following equation:

% scavenging = [(Ac − A)/(Ac − Ab)] × 100, where Ac is the absorbance of the control, Ab is the absorbance of the blank, and A is the absorbance of the sample.

#### 3.4.3. Copper (Cu^2+^) Chelation Assay

The copper chelation assay is used to assess the ability of compounds to complex transition metal ions, particularly Cu^2+^, thereby limiting their participation in redox reactions that promote the formation of reactive oxygen species. Through this mechanism, metal ion sequestration reduces the catalytic activity of copper in Fenton-type reactions, representing a preventive antioxidant mechanism that limits oxidative processes prior to the generation of free radicals.

The copper-chelating activity was evaluated according to the procedure described by Megías et al. [58]. The assay was carried out in a microplate format, where sample solutions (1.0 mg/mL) were first dispensed into the wells, followed by the sequential addition of pyrocatechol violet and copper (II) sulfate pentahydrate. The reaction mixture was homogenized after each reagent addition to ensure proper mixing. Absorbance was subsequently recorded at 632 nm using a microplate reader. EDTA (ethylenediaminetetraacetic acid) was employed as the reference chelating agent. The results were expressed as the percentage of copper chelation, calculated according to the following equation:

% chelation = [(Ab − Aa)/Ab] × 100, where Ab is the absorbance of the blank and Aa is the absorbance of the sample.

#### 3.4.4. DPPH Assay

The DPPH radical scavenging assay determines the capacity of antioxidant compounds to neutralize the stable free radical 2,2-diphenyl-1-picrylhydrazyl (DPPH•) through electron or hydrogen atom transfer reactions. This interaction leads to a reduction in the characteristic absorbance of the DPPH radical, reflecting the radical-scavenging potential of the tested compounds. The method is commonly applied to evaluate the antioxidant activity of low-molecular-weight and soluble substances. Ascorbic acid was employed as the reference antioxidant compound.

The assay was conducted in a 96-well microplate following the procedure described by Cheng et al. [59]. Briefly, 100 µL of the sample solution (0.5 mg/mL in distilled water) was mixed with 100 µL of a methanolic DPPH solution (150 μM) and the mixture was shaken for 60 s to ensure homogenization. The reaction was then allowed to proceed for 30 min in the dark at room temperature. After this period, absorbance was recorded at 515 nm using a microplate reader (Epoch, BioTek Instruments, Winooski, VT, USA). The percentage of DPPH radical scavenging activity was calculated using the equation presented above, where Asample corresponds to the absorbance of the reaction mixture containing the sample and DPPH solution after 30 min, Ablank represents the absorbance of ethanol (200 µL), and Acontrol corresponds to the absorbance of the DPPH solution alone (200 µL) after 30 min.

DPPH scavenging (%) = 100 − {[(Asample − Ablank) × 100]/(Acontrol)}

#### 3.4.5. Hydrogen Peroxide Scavenging Assay

The hydrogen peroxide scavenging assay is employed to determine the capacity of antioxidant compounds to eliminate hydrogen peroxide (H_2_O_2_), a moderately stable reactive oxygen species that can diffuse through biological membranes and act as a precursor for more reactive radicals in the presence of transition metals. Consequently, the removal of hydrogen peroxide contributes to limiting oxidative stress and preventing the formation of highly reactive oxygen species in biological environments.

The antioxidant activity was evaluated using a modified procedure based on the method described by Jesumani et al. [60]. A 40 mM hydrogen peroxide solution was prepared in 100 mM sodium phosphate buffer (pH 7.4). For the assay, 400 μL of the sample solution (1 mg/mL) were combined with 600 μL of the hydrogen peroxide solution in hemolysis tubes, and the mixtures were homogenized and incubated for 10 min at room temperature in the absence of light.

Sodium phosphate buffer was used as the negative control, whereas the hydrogen peroxide solution prepared in the same buffer served as the positive control. Prior to evaluating the scavenging activity, UV–Vis spectra of all samples were recorded between 190 and 700 nm under identical experimental conditions to verify whether the samples presented intrinsic absorbance at the detection wavelength (230 nm).

Because the samples showed measurable absorbance at 230 nm, all measurements were corrected using appropriate sample blanks. The blank solution contained the same buffer and sample at the corresponding concentration but without hydrogen peroxide. The absorbance values obtained for these blanks were subtracted from the respective reaction readings, thereby eliminating spectral interference caused by the intrinsic absorbance of the samples. Ascorbic acid was used as the reference antioxidant compound.

Hydrogen peroxide scavenging activity was quantified by spectrophotometric measurement at 230 nm, considering the absorbance of the positive control as representing 0% scavenging activity. The results were expressed as the percentage of hydrogen peroxide scavenging relative to positive control.

### 3.5. Cytotoxicity Assay in Mammalian Cell Lines

The cytotoxic potential of *L. alba* essential oils obtained from dry-season (A1T–DS) and rainy-season (A5T–RS) samples was evaluated using five mammalian cell lines: murine fibroblasts NIH/3T3 (ATCC^®^ CRL-1658™), murine fibroblasts L929 (ATCC^®^ CCL-1™), Chinese hamster ovary cells CHO-K1 (ATCC^®^ CCL-61™), human cervical carcinoma cells HeLa (ATCC^®^ CCL-2™), and human hepatocellular carcinoma cells HepG2 (ATCC^®^ HB-8065™).

Cells were cultured in Dulbecco’s Modified Eagle Medium (DMEM, high glucose) supplemented with 10% fetal bovine serum (FBS), 1% penicillin–streptomycin (100 U/mL penicillin and 100 µg/mL streptomycin), and 2 mM L-glutamine. CHO-K1 cells were maintained in Ham’s F-12 medium supplemented with 10% FBS and antibiotics. All cell lines were incubated at 37 °C in a humidified atmosphere containing 5% CO_2_. Cells were subcultured using trypsin–EDTA solution when reaching approximately 80% confluence.

Essential oils were dissolved in dimethyl sulfoxide (DMSO) to obtain stock solutions. Working concentrations were prepared by diluting the stocks in culture medium immediately before use. The final DMSO concentration in all treatments was maintained at ≤0.1% (*v*/*v*), including vehicle control groups.

Cytotoxicity was evaluated using the colorimetric MTT assay to determine the effect of the samples on cell metabolic activity. Cells were plated in 96-well culture plates at a density of 1 × 10^4^ cells per well and maintained for 24 h to permit cell adhesion. After this period, the culture medium was replaced with fresh medium containing the essential oils at concentrations ranging from 1 to 200 µg/mL. The cells were then incubated with the treatments for 24 h under standard cell culture conditions.

Following the exposure period, 20 µL of MTT solution (5 mg/mL prepared in culture medium) was added to each well, and the plates were incubated for an additional 4 h at 37 °C to allow the formation of formazan crystals by metabolically active cells. Subsequently, the supernatant was carefully discarded, and the insoluble formazan crystals were dissolved by adding 150 µL of methanol to each well. The optical density was then measured at 570 nm using a microplate reader.

Cell cytotoxicity was expressed as the percentage of MTT reduction relative to untreated control cells. The half-maximal inhibitory concentration (IC_50_) values were calculated from concentration–response curves by nonlinear regression analysis using the viability data obtained from the treatments.

### 3.6. Cytoprotective Assay Against Hydrogen Peroxide-Induced Oxidative Stress

The cytoprotective activity of *L. alba* essential oils was investigated using L929 murine fibroblast cells exposed to oxidative stress induced by hydrogen peroxide. Cells were plated in 96-well culture plates at a density of 1 × 10^4^ cells per well and maintained for 24 h to allow proper cell adhesion. After this period, the cells were pretreated with essential oil samples at concentrations of 5, 10, and 25 µg/mL for 24 h. Subsequently, oxidative stress was induced by treating the cells with hydrogen peroxide (H_2_O_2_) at a final concentration of 300 µM for 2 h. Following peroxide exposure, the culture medium was removed and replaced with fresh medium prior to evaluation of cell viability using the MTT assay.

Essential oils obtained from dry-season (A1T–DS) and rainy-season (A5T–RS) samples were initially dissolved in dimethyl sulfoxide (DMSO) to generate stock solutions. These stock preparations were subsequently diluted in culture medium to obtain working concentrations of 5, 10, and 25 µg/mL. The final concentration of DMSO in all experimental conditions was kept below 0.1% (*v*/*v*), including the vehicle control groups. Cell viability was determined by MTT reduction and expressed as a percentage relative to untreated control cells.

### 3.7. Intracellular Reactive Oxygen Species (ROS) Assay (DCFH-DA)

Intracellular reactive oxygen species (ROS) generation was assessed using the fluorescent probe 2′,7′-dichlorodihydrofluorescein diacetate (DCFH-DA), according to previously reported procedures with slight modifications [61].

L929 murine fibroblast cells were plated in 96-well plates at a density of 1 × 10^4^ cells per well and maintained for 24 h to ensure adequate cell adhesion. After this period, the culture medium was replaced and the cells were pretreated for 24 h with essential oils derived from the dry-season sample (A1T–DS) or the rainy-season sample (A5T–RS) at final concentrations of 5, 10, and 25 µg/mL. Vehicle control wells received culture medium containing 0.1% DMSO, which did not produce detectable cytotoxic effects under the experimental conditions.

Following the pretreatment period, oxidative stress was induced by exposing the cells to hydrogen peroxide (H_2_O_2_) at a final concentration of 300 µM for 2 h. Subsequently, cells were washed with phosphate-buffered saline (PBS) and incubated with DCFH-DA (10 µM) prepared in serum-free DMEM for 30 min at 37 °C in the dark.

After incubation, the probe-containing solution was discarded, and the cells were gently rinsed with PBS to remove residual extracellular probe. Intracellular ROS levels were quantified by measuring fluorescence intensity using a microplate reader, with excitation and emission wavelengths set at 485 nm and 535 nm, respectively.

The fluorescence readings were normalized to untreated control cells and expressed as a percentage of intracellular ROS production. All experiments were conducted in triplicate, and the data were reported as mean ± standard deviation (SD). The H_2_O_2_ concentration (300 µM) was selected based on preliminary assays and previous literature reports to induce oxidative stress while avoiding excessive cell mortality.

### 3.8. Statistical Analyses

Experiments were performed in triplicate, with three independent experimental repetitions. Results were expressed as mean ± standard deviation (SD). Statistical comparisons were performed using two-way analysis of variance (ANOVA), followed by Tukey’s post hoc test to evaluate differences among treatments. Statistical significance was considered at *p* < 0.05.

Principal component analysis (PCA) was applied as an exploratory multivariate statistical method to evaluate patterns of similarity and chemical variability among *L. alba* essential oil samples based on their chemical composition. The PCA was performed using the relative percentages of the identified compounds obtained by GC–MS analysis.

Prior to analysis, the data matrix was standardized by autoscaling (mean-centered and scaled to unit variance) to minimize the influence of differences in magnitude among variables and to ensure that all compounds contributed equally to the model. The covariance structure of the standardized dataset was then decomposed to obtain the principal components. The first two principal components (PC1 and PC2) were selected for interpretation based on the amount of explained variance and were used to construct score and loading plots. The PCA results were visualized using biplots to simultaneously assess sample distribution and variable contributions.

To quantitatively assess chemical complexity, the Shannon diversity index (H′) was calculated for each essential oil sample based on the relative abundance (p_i_) of identified constituents, according to the equation H′ = −∑(p_i_ ln p_i_), where p_i_ represents the proportional contribution of each compound. This index was used as a metric of compositional diversity, reflecting both richness and evenness of chemical constituents. The diversity analysis was applied as a complementary chemometric approach to support interpretation of compositional differences in relation to biological activity.

Statistical computations and data visualization were performed using the Python programming language (version 3.x, Python Software Foundation, Wilmington, DE, USA). Multivariate computations (PCA) were implemented using the Scikit-learn library (version 1.3, INRIA, Le Chesnay-Rocquencourt, France), while data handling and visualization were supported by Pandas (version 2.0) and Matplotlib version 3.6 (NumFOCUS, Austin, TX, USA).

## 4. Conclusions

This study demonstrates that *L. alba* essential oils maintain a stable citral chemotype across seasons, as evidenced by PCA, indicating that seasonal variation does not compromise the dominant chemical fingerprint. However, quantitative modulation of secondary metabolites significantly dictates biological performance. The rainy-season oil (A5T–RS) exhibited markedly higher chemical diversity (H′ = 1.94 vs. 0.82 for A1T–DS), characterized by an enrichment in oxygenated monoterpenes such as geraniol and nerolidol.

Functionally, A5T–RS displayed superior multi-target antioxidant capacity and enhanced intracellular ROS reduction, promoting superior redox homeostasis at the cellular level. Furthermore, this seasonal enrichment of minor metabolites was strongly correlated with increased selective antiproliferative activity against tumor-derived cell lines. Collectively, these findings indicate that the bioactivity of *L. alba* essential oil is not determined solely by chemotype identity, but rather by the seasonal modulation of chemical diversity and compositional balance. These results underscore the pivotal role of ecological factors in shaping phytochemical complexity and optimizing the therapeutic potential of aromatic plants.

## Figures and Tables

**Table 1:** Yield of essential oils obtained from *L. alba* Mill. leaves collected over different months and seasons.

Sample	Fresh Plant Material Mass (g)	Essential Oil Mass (g)	Yield (%)
A1T-DS	39.432	0.123	0.330
A2M-DS	47.604	0.180	0.379
A3T-DS	41.155	0.115	0.279
A4M-DS	39.298	0.188	0.479
A5T-RS	40.944	0.029	0.070
A6M-RS	40.019	0.109	0.273
A7T-DS	40.622	0.255	0.628
A8M-DS	40.098	0.172	0.429
A9T-DS	40.557	0.190	0.469

DS: dry season; RS: rainy season; A: sample; M: morning; T: afternoon; numbers indicate the collection month.

**Table 2:** Chemical composition of the essential oil from *L. alba*.

	IR	A1T-DS	A2M-DS	A3T-DS	A4M-DS	A5T-RS	A6M-RS	A7T-DS	A8M-DS	A9T-DS
5-hepten-2-one	938	-	1.86	2.58	2.73	8.18	4.36	2.37	2.27	-
β-myrcene	958	2.54	2.10	2.34	2.54	4.57	1.33	3.34	3.51	2.37
1-octen-3-ol	969	-	1.47	1.76	1.89	1.44	1.19	1.79	1.56	4.16
Eucalyptol	1059	-	-	1.06	-	-	-	-	-	-
D-limonene	1018	5.46	4.84	5.78	7.55	8.02	2.58	6.66	6.75	4.73
Linalool	1082	-	1.08	0.78	-	1.28	1.35	0.99	0.95	1.15
Citronellal	1125	-	-	-	-	1.14	0.59	0.34	-	-
Estragole	1172	-	-	1.91	-	-	-	-	-	-
Citral	1174	80.69	68.78	62.91	66.97	42.32	57.41	62.32	59.27	65.94
Citronellol	1179	-	-	-	-	-	4.22		-	-
Carvone	1190	3.40	2.75	3.80	4.61	4.69	3.94	3.26	11.18	3.80
Geraniol	1228	3.85	14.19	7.17	12.31	14.06	9.99	14.86	16.65	8.22
Geranyl acetate	1352	1.70	1.43	0.95	1.40	1.17	0.71	1.16	1.71	1.12
Germacrene D	1515	2.35	-	-	-	1.72	-	-	1.11	-
Nerolidol	1564	-	0.67	0.53	-	3.28	2.56	1.03	0.97	1.05
Phytol	2045	-	-	-	-	-	0.51	-	-	-

A1T-DS, A3T-DS, and A9T-DS correspond to samples collected in the afternoon during the dry season; A2M-DS and A4M-DS to samples collected in the morning during the dry season; A5T-RS and A7T-RS to samples collected in the afternoon during the rainy season; A6M-RS and A8M-RS samples collected in the morning during the rainy season. DS, dry season; RS, rainy season; M, morning; T, afternoon.

**Table 3:** In vitro antioxidant activity of *L. alba* essential oils collected during dry (A1T–DS) and rainy (A5T–RS) seasons at different concentrations.

Sample	mg/mL	DPPH (%)	Reducing Power (%)	H_2_O_2_ Scavenging (%)	Total Antioxidant Capacity (TAC) *
A1T–DS	0.5	23.63 ± 1.70 ^a^	43.67 ± 1.53 ^a^	41.67 ± 2.08 ^a^	23.67 ± 1.53 ^a^
A1T–DS	1.0	22.70 ± 1.44 ^a^	70.67 ± 1.15 ^b^	69.00 ± 1.00 ^b^	35.33 ± 0.58 ^b^
A5T–RS	0.5	23.07 ± 0.93 ^a^	43.00 ± 2.00 ^a^	44.33 ± 1.15 ^a^	23.67 ± 1.53 ^a^
A5T–RS	1.0	48.33 ± 4.73 ^b^	93.67 ± 1.53 ^c^	91.67 ± 1.53 ^c^	55.00 ± 3.00 ^c^
Cathequin	0.5	94.38 ± 0.29 ^c^	186.72 ± 6.18 ^d^	51.23 ± 1.53 ^d^	387.23 ± 4.70 ^d^
Cathequin	1.0	94.30 ± 5.84 ^c^	243.96 ± 30.40 ^d^	75.95 ± 5.23 ^b^	691.24 ± 5.26 ^d^

Results are expressed as mean ± SD. Different letters within the same column indicate statistically significant differences (*p* < 0.05). * Total antioxidant capacity (TAC) is expressed as ascorbic acid equivalents per gram of sample. Catechin was included as a reference antioxidant control to validate assay performance and to provide a methodological benchmark for radical scavenging and redox-related measurements.

**Table 4:** Cytotoxicity (IC50, µg/mL) and Selectivity Index (SI) of *L. alba* Essential Oils.

Cell Line	Cell Type	A1T–DS (IC_50_)	A5T–RS (IC_50_)
NIH/3T3	Murine Fibroblast	>100 ^a^	85.21 ± 2.31 ^b^
L929	Murine Fibroblast	>120 ^a^	95.32 ± 4.37 ^b^
CHO-K1	Hamster Ovary	80.09 ± 2.29 ^a^	65.23 ± 3.71 ^b^
HeLa	Human Cervical Tumor	55.23 ± 5.51 ^a^	35.36 ± 1.29 ^b^
HepG2	Human Hepatocarcinoma	70.21 ± 3.43 ^a^	45.52 ± 2.52 ^b^
SI (HeLa) *	Selectivity Index	>1.82 ^a^	2.43 ± 0.10 ^b^
SI (HepG2) *	Selectivity Index	>1.43 ^a^	1.89 ± 0.11 ^b^

IC_50_ values represent the concentration (µg/mL) required to inhibit 50% of cell growth and are expressed as mean ± standard deviation (SD) from independent experimental triplicates. * SI was calculated as the ratio of IC_50_ (NIH/3T3)/IC_50_ (Tumor line). For calculations where IC_50_ exceeded the maximum tested concentration (e.g., >100 or >120 µg/mL), the limit value was used to provide a conservative estimate of the Selectivity Index (SI). In these instances, SD calculation and formal statistical comparison were not applicable as the true IC_50_ lies beyond the experimental range. For all other values, different letters (a, b) within the same row indicate significant statistical differences between seasonal samples (A1T–DS vs. A5T–RS) according to Tukey’s post hoc test (*p* < 0.05).

**Table 5:** Protective effect of *L. alba* Essential Oils against H_2_O_2_-induced cytotoxicity in L929 cells.

Sample	mg/mL	MTT Reduction (%)
NC	-	100 ^a^
H_2_O_2_ (300 µM)	-	47.4 ± 4.1 ^b^
A1T–DS	25	101 ± 2.1 ^a^
A5T–RS	25	98.5 ± 5.1 ^a^
A1T–DS + H_2_O_2_	5.0	58.5 ± 6.1 ^c^
A1T–DS + H_2_O_2_	10.0	62.5 ± 4.8 ^d^
A1T–DS + H_2_O_2_	25.0	65.6 ± 5.3 ^e^
A5T–RS + H_2_O_2_	5.0	68.8 ± 5.1 ^d,e^
A5T–RS + H_2_O_2_	10.0	78.4 ± 4.3 ^f^
A5T–RS + H_2_O_2_	25.0	86.3 ± 3.1 ^a^

Values are expressed as mean ± standard deviation. Means sharing the same letter within a column are not significantly different according to Tukey’s multiple comparison test (*p* < 0.05). NC: negative control (medium only).

**Table 6:** Seasonal variation in the protective effects of *L. alba* essential oils against H_2_O_2_-induced ROS generation in L929 fibroblasts.

Sample	mg/mL	ROS (%)
NC	-	100 ±3.9 ^a^
H_2_O_2_ (300 µM)	-	195.0 ± 10.1 ^b^
A1T–DS	25	101.2 ± 2.1 ^a^
A5T–RS	25	103.2 ± 3.5 ^a^
A1T–DS + H_2_O_2_	5.0	175.2 ± 10.5 ^b,c^
A1T–DS + H_2_O_2_	10.0	168.3 ± 7.0 ^c^
A1T–DS + H_2_O_2_	25.0	148.3 ± 8.2 ^d^
A5T–RS + H_2_O_2_	5.0	159.3 ± 9.3 ^c^
A5T–RS + H_2_O_2_	10.0	135.3 ± 7.6 ^f^
A5T–RS + H_2_O_2_	25.0	115.2 ± 7.3 ^g^

Values are expressed as mean ± standard deviation. Means sharing the same letter within a column are not significantly different according to Tukey’s multiple comparison test (*p* < 0.05). NC: negative control (medium only).

## Data Availability

Representative GC–MS total ion chromatograms (TIC) and mass spectra used for compound identification are provided in the Supplementary Material. The complete dataset, including chromatograms and spectra for all samples as well as the raw optical density (OD) values from the cell-based assays, is available from the corresponding author upon reasonable request.

## Supplementary Materials

The following supporting information can be downloaded at: https://www.mdpi.com/article/10.3390/molecules31061035/s1, Figure S1: Representative GC–MS total ion chromatograms (TIC) and mass spectra used for compound identification in A1T-DS.

## Abbreviations

The following abbreviations are used in this manuscript:

A1T–DS	First month afternoon dry season sample
A5T–RS	Fifth month afternoon rainy season sample
ANOVA	Analysis of variance
CHO	Chinese hamster ovary cells
DB-1	Dimethylpolysiloxane
DCFH-DA	Diacetyldichlorofluorescein
DMEM	Dulbecco’s Modified Eagle Medium
DMSO	Dimethyl Sulfoxide
DPPH	2,2-diphenyl-1-picrylhydrazyl
DS	Dry season
EDTA	Ethylenediamine tetraacetic acid
EI	Electron impact
FBS	Fetal bovine serum
GC-MS	Gas Chromatography–Mass Spectrometry
HeLa	Human cervical carcinoma cells
HepG2	Human hepatocellular carcinoma cells
IC	Inhibitory concentration
L929	Murine fibroblasts
M	Morning
MAPK	Mitogen-Activated Protein Kinases
MEP	Methylerythritol phosphate pathway
MTT	3-(4,5-dimethylthiazol-2-yl)-2,5-diphenyltetrazolium bromide
NC	Negative control
NIH/3T3	Murine fibroblasts
P53	Tumor protein p53
PBS	Phosphate-buffered saline
PC1	First principal component
PC2	Second principal component
PCA	Principal component analysis
ROS	Reactive oxygen species
RS	Rainy season
SD	Standard deviation
SI	Selectivity index
SISGEN	National System for the Management of Genetic Heritage and Associated Traditional Knowledge
STAT3	Signal Transducer and Activator of Transcription 3
T	Afternoon
TAC	Total antioxidant capacity
TCA	Trichloroacetic
