# Supplementary material for: Impact of Dry and Rainy Seasons on the Chemical Profile and Antioxidant Activity of Lippia alba Essential Oil
Source: Molecules. 2026 Mar 20;31(6):1035. doi: 10.3390/molecules31061035 (PMC13029229; doi:10.3390/molecules31061035)
Supplement: Supplementary file 1 [file molecules-31-01035-s001.zip › molecules-4173627-supplementary.pdf]

Figure S1 - Representative GC–MS total ion chromatograms (TIC) and mass spectra used for compound identification in A1T-DS. The complete dataset, including chromatograms and spectra for all samples as well as the raw optical density (OD) values from the cell-based assays, is available from the corresponding author upon reasonable request.

GCMS Sample Information

Sample Name : A1T-DS
Operator : Admin
Acquisition Date : 20/11/2023 18:29:00

Method File : C:\GCMSsolution\Data\Joao Carlos\ÓLEO ESSENCIAL-JC.qgm
Tuning File : C:\GCMSsolution\System\Tune1\tuning 01-08-2023.qgt
Modified : 28/11/2023 09:17:16

Chromatogram AMOSTRA1 C:\GCMSsolution\Data\DANIELE-CHICO\OE\RENATA-UFRN\AMOSTRA1.qgd

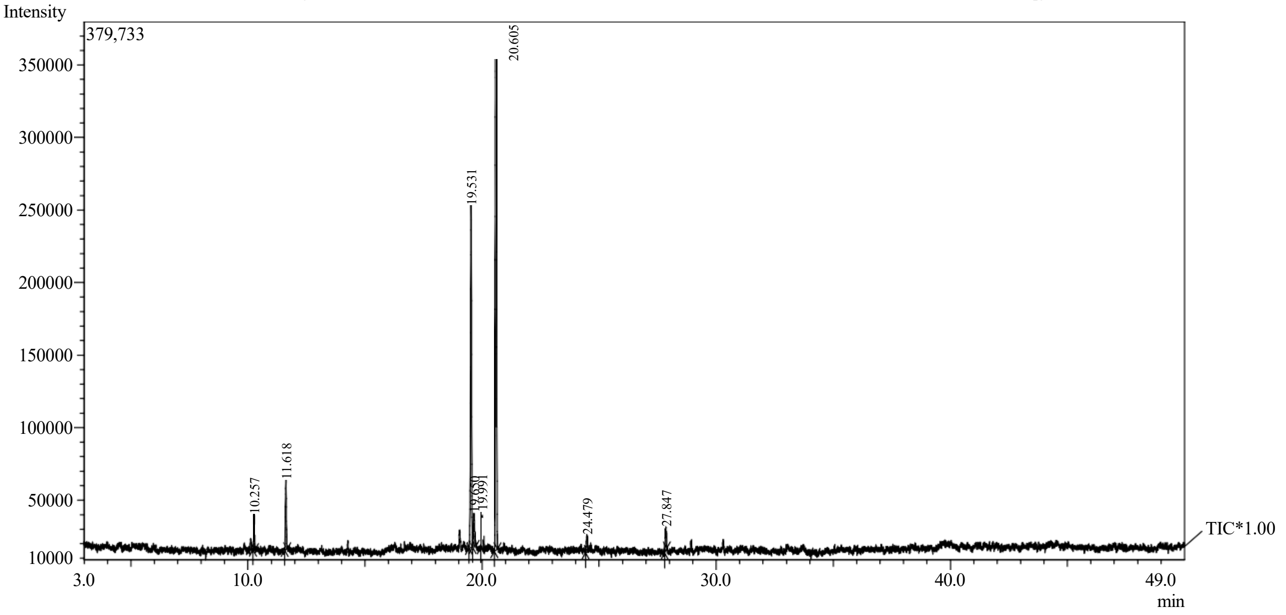

GCMS Method

Column Information: DB-1 (30 m x 0,25 mm id x 0,25 um film)
Inj. Initial Temp. :25.00 °C Interface Temp. :230.00 °C Control Mode :Split Column Inlet Pressure :100.0 kPa
Column Flow :1.7 mL/min Linear Velocity :47.4 cm/sec Split Ratio :27 Total Flow :50.0 mL/min Equilibrium Time :1.00 min
Oven Temp. Program :Rate Temperature(°C) Keep Time(min)
- 50.0 0.00

GCMS Peak Report TIC

Peak#	R.Time	I.Time	Area	Height	A/H	Mark	Name	Base m/z	Area%	ht%
1	10.257	10.210	58035	24546	2.36		.beta.-Myrcene	41.05	2.54	3.39
2	11.618	11.565	124697	47726	2.61		D-Limonene	68.05	5.46	6.59
3	19.531	19.445	721615	236020	3.06		2,6-Octadienal, 3,7-dimethyl-, (Z)-	41.05	31.60	2.58
4	19.650	19.590	77730	23519	3.30	V	2-Cyclohexen-1-one, 2-methyl-5-(1-methylethe	82.00	3.40	3.25
5	19.991	19.950	87854	26166	3.36	S	2,6-Octadien-1-ol, 3,7-dimethyl-, (E)-	69.05	3.85	3.61
6	20.605	20.525	1120990	338378	3.31	V	2,6-Octadienal, 3,7-dimethyl-	41.05	49.09	6.70
7	24.479	24.415	38897	11893	3.27		2,6-Octadien-1-ol, 3,7-dimethyl-, acetate, (E)-	69.05	1.70	1.64
8	27.847	27.800	53630	16288	3.29	V	1,6-Cyclodecadiene, 1-methyl-5-methylene-8-(1	161.00	2.35	2.25
			2283448	724536					100.00	0.00

GCMS Library

<< Target >>
Line#:1 R.Time:10.255(Scan#:1452) MassPeaks:255 BasePeak:41.05(5293)
RawMode:Averaged 10.250-10.260(1451-1453) BG Mode:Calc. from Peak

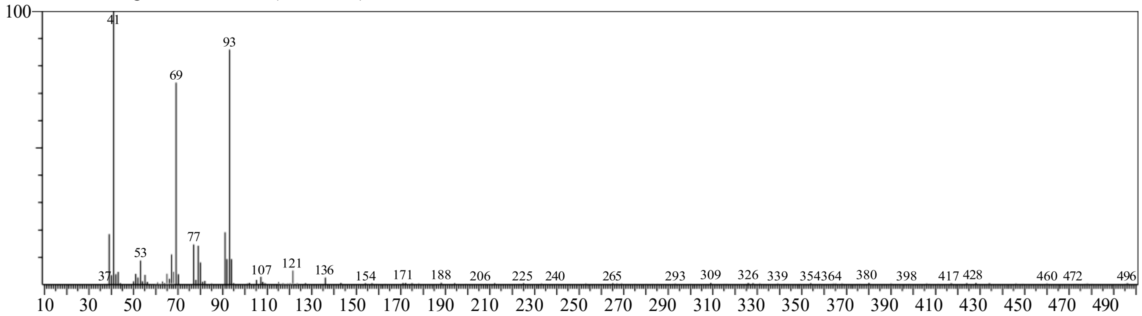

Hit#:1 Entry:9689 Library:NIST08.LIB
SI:95 Formula:C10H16 CAS:123-35-3 MolWeight:136 RetIndex:958
CompName:.beta.-Myrcene $$ 1,6-Octadiene, 7-methyl-3-methylene- $$ Myrcene $$ 7-Methyl-3-methylene-1,6-octadiene $$ 7-Methyl-3-methyleneoctadie

Hit#:2 Entry:6401 Library:NIST08s.LIB
SI:94 Formula:C10H16 CAS:123-35-3 MolWeight:136 RetIndex:958
CompName:.beta.-Myrcene $$ 1,6-Octadiene, 7-methyl-3-methylene- $$ Myrcene $$ 7-Methyl-3-methylene-1,6-octadiene $$ 7-Methyl-3-methyleneoctadie

Hit#:3 Entry:6429 Library:NIST08s.LIB
SI:93 Formula:C10H16 CAS:123-35-3 MolWeight:136 RetIndex:958
CompName:.beta.-Myrcene $$ 1,6-Octadiene, 7-methyl-3-methylene- $$ Myrcene $$ 7-Methyl-3-methylene-1,6-octadiene $$ 7-Methyl-3-methyleneoctadie

Hit#:4 Entry:6426 Library:NIST08s.LIB
SI:89 Formula:C10H16 CAS:127-91-3 MolWeight:136 RetIndex:943
CompName:.beta.-Pinene $$ Bicyclo[3.1.1]heptane, 6,6-dimethyl-2-methylene- $$ 2(10)-Pinene $$ Nopinen $$ Nopinene $$ Pseudopinen $$ Pseudopinene

Hit#:5 Entry:6428 Library:NIST08s.LIB
SI:89 Formula:C10H16 CAS:127-91-3 MolWeight:136 RetIndex:943
CompName:.beta.-Pinene $$ Bicyclo[3.1.1]heptane, 6,6-dimethyl-2-methylene- $$ 2(10)-Pinene $$ Nopinen $$ Nopinene $$ Pseudopinen $$ Pseudopinene

<< Target >>
Line#:2 R.Time:11.620(Scan#:1725) MassPeaks:278 BasePeak:68.05(7542)
RawMode:Averaged 11.615-11.625(1724-1726) BG Mode:Calc. from Peak

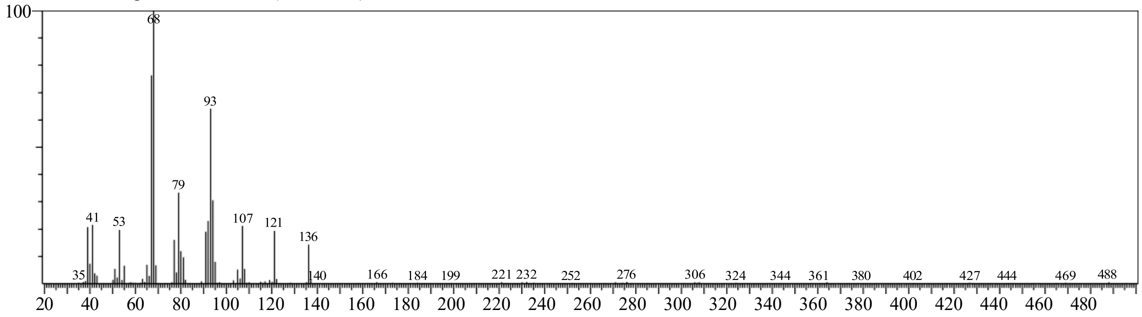

Hit#:1 Entry:6414 Library:NIST08s.LIB
SI:94 Formula:C10H16 CAS:5989-27-5 MolWeight:136 RetIndex:1018
CompName:D-Limonene $$ Cyclohexene, 1-methyl-4-(1-methylethenyl)-, (R)- $$ p-Mentha-1,8-diene, (R)-(+)- $$ (+)-(R)-Limonene $$ (+)-(4R)-Limonen

Hit#:2 Entry:6413 Library:NIST08s.LIB
SI:94 Formula:C10H16 CAS:5989-27-5 MolWeight:136 RetIndex:1018
CompName:D-Limonene $$ Cyclohexene, 1-methyl-4-(1-methylethenyl)-, (R)- $$ p-Mentha-1,8-diene, (R)-(+)- $$ (+)-(R)-Limonene $$ (+)-(4R)-Limonen

Hit#:3 Entry:9719 Library:NIST08.LIB
SI:94 Formula:C10H16 CAS:5989-27-5 MolWeight:136 RetIndex:1018
CompName:D-Limonene $$ Cyclohexene, 1-methyl-4-(1-methylethenyl)-, (R)- $$ p-Mentha-1,8-diene, (R)-(+)- $$ (+)-(R)-Limonene $$ (+)-(4R)-Limonen

Hit#:4 Entry:6410 Library:NIST08s.LIB
SI:93 Formula:C10H16 CAS:5989-54-8 MolWeight:136 RetIndex:1018
CompName:Cyclohexene, 1-methyl-4-(1-methylethenyl)-, (S)- $$ p-Mentha-1,8-diene, (S)-(-)- $$ (-)-Limonene $$ L-Limonene $$ Limonene $$ 4-Isoprope

Hit#:5 Entry:9718 Library:NIST08.LIB
SI:92 Formula:C10H16 CAS:19465-02-2 MolWeight:136 RetIndex:934
CompName:Cyclobutane, 1,2-bis(1-methylethenyl)-, trans- $$ 1,2-Diisopropenylcyclobutane # $$

<< Target >>
Line#:3 R.Time:19.530(Scan#:3307) MassPeaks:278 BasePeak:41.05(45853)
RawMode:Averaged 19.525-19.535(3306-3308) BG Mode:Calc. from Peak

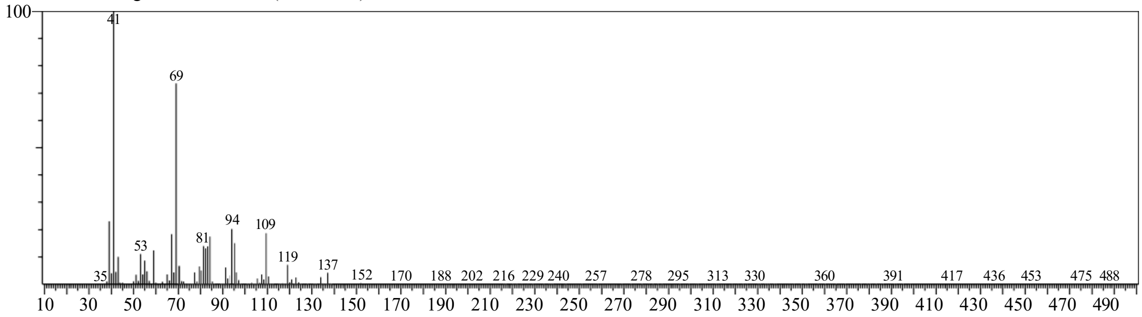

Hit#:1 Entry:9118 Library:NIST08s.LIB
SI:95 Formula:C10H16O CAS:106-26-3 MolWeight:152 RetIndex:1174
CompName:2,6-Octadienal, 3,7-dimethyl-, (Z)- $$ .beta.-Citral $$ cis-Citral $$ cis-3,7-Dimethyl-2,6-octadienal $$ Citral b $$ Neral $$ Z-Citral $$ (Z)-3,7-

Hit#:2 Entry:9150 Library:NIST08s.LIB
SI:95 Formula:C10H16O CAS:106-26-3 MolWeight:152 RetIndex:1174
CompName:2,6-Octadienal, 3,7-dimethyl-, (Z)- $$ .beta.-Citral $$ cis-Citral $$ cis-3,7-Dimethyl-2,6-octadienal $$ Citral b $$ Neral $$ Z-Citral $$ (Z)-3,7-

Hit#:3 Entry:16176 Library:NIST08.LIB
SI:92 Formula:C10H16O CAS:106-26-3 MolWeight:152 RetIndex:1174
CompName:2,6-Octadienal, 3,7-dimethyl-, (Z)- $$ .beta.-Citral $$ cis-Citral $$ cis-3,7-Dimethyl-2,6-octadienal $$ Citral b $$ Neral $$ Z-Citral $$ (Z)-3,7-

Hit#:4 Entry:9121 Library:NIST08s.LIB
SI:92 Formula:C10H16O CAS:5392-40-5 MolWeight:152 RetIndex:1174
CompName:2,6-Octadienal, 3,7-dimethyl- $$ Citral $$ 3,7-Dimethyl-2,6-octadienal $$ Citral,c&t $$ cis,trans-Citral $$ Geranial $$ NCI-C56348 $$ 3,7-Di

Hit#:5 Entry:9147 Library:NIST08s.LIB
SI:92 Formula:C10H16O CAS:5392-40-5 MolWeight:152 RetIndex:1174
CompName:2,6-Octadienal, 3,7-dimethyl- $$ Citral $$ 3,7-Dimethyl-2,6-octadienal $$ Citral,c&t $$ cis,trans-Citral $$ Geranial $$ NCI-C56348 $$ 3,7-Di

<< Target >>
Line#:4 R.Time:19.650(Scan#:3331) MassPeaks:295 BasePeak:82.00(3695)
RawMode:Averaged 19.645-19.655(3330-3332) BG Mode:Calc. from Peak

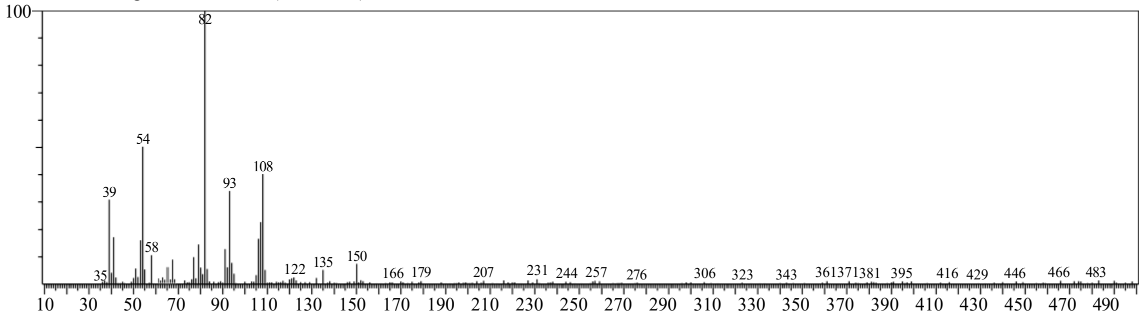

Hit#:1 Entry:15239 Library:NIST08.LIB
SI:93 Formula:C10H14O CAS:2244-16-8 MolWeight:150 RetIndex:1190
CompName:2-Cyclohexen-1-one, 2-methyl-5-(1-methylethenyl)-, (S)- $$ p-Mentha-6,8-dien-2-one, (S)-(+)- $$ (+)-Carvone $$ (S)-(+)-Carvone $$ (S)-Car

Hit#:2 Entry:15241 Library:NIST08.LIB
SI:93 Formula:C10H14O CAS:6485-40-1 MolWeight:150 RetIndex:1190
CompName:2-Cyclohexen-1-one, 2-methyl-5-(1-methylethenyl)-, (R)- $$ p-Mentha-6,8-dien-2-one, (R)-(-)- $$ (-)-(R)-Carvone $$ (-)-p-Mentha-6,8-dien-2

Hit#:3 Entry:8681 Library:NIST08s.LIB
SI:92 Formula:C10H14O CAS:2244-16-8 MolWeight:150 RetIndex:1190
CompName:2-Cyclohexen-1-one, 2-methyl-5-(1-methylethenyl)-, (S)- $$ p-Mentha-6,8-dien-2-one, (S)-(+)- $$ (+)-Carvone $$ (S)-(+)-Carvone $$ (S)-Car

Hit#:4 Entry:15240 Library:NIST08.LIB
SI:92 Formula:C10H14O CAS:99-49-0 MolWeight:150 RetIndex:1190
CompName:2-Cyclohexen-1-one, 2-methyl-5-(1-methylethenyl)- $$ p-Mentha-6,8-dien-2-one $$ Carvol $$ Carvone $$ Karvon $$ 1-Carvone $$ .delta.(sup

Hit#:5 Entry:8682 Library:NIST08s.LIB
SI:92 Formula:C10H14O CAS:99-49-0 MolWeight:150 RetIndex:1190
CompName:2-Cyclohexen-1-one, 2-methyl-5-(1-methylethenyl)- $$ p-Mentha-6,8-dien-2-one $$ Carvol $$ Carvone $$ Karvon $$ 1-Carvone $$ .delta.(sup

<< Target >>
Line#:5 R.Time:19.990(Scan#:3399) MassPeaks:265 BasePeak:69.05(6248)
RawMode:Averaged 19.985-19.995(3398-3400) BG Mode:Calc. from Peak

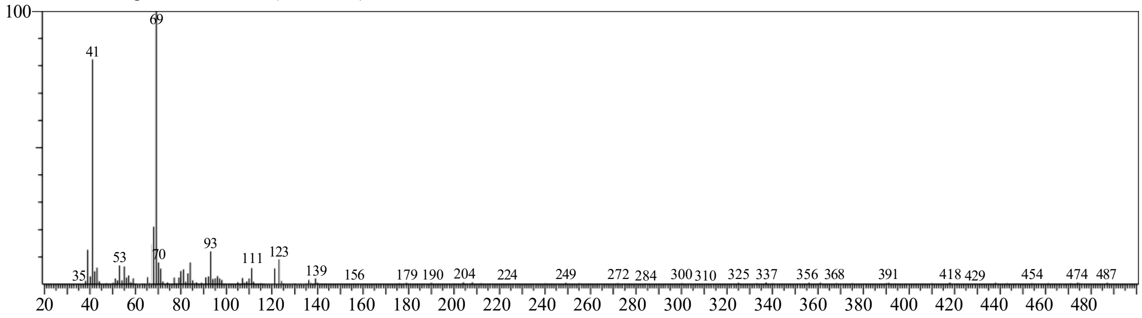

Hit#:1 Entry:17359 Library:NIST08.LIB
SI:95 Formula:C10H18O CAS:106-24-1 MolWeight:154 RetIndex:1228
CompName:2,6-Octadien-1-ol, 3,7-dimethyl-, (E)- $$ trans-Geraniol $$ Guaniol $$ Lemonol $$ Nerol $$ Neryl Alcohol $$ Geraniol $$ trans-3,7-Dimethyl

Hit#:2 Entry:9597 Library:NIST08s.LIB
SI:94 Formula:C10H18O CAS:106-24-1 MolWeight:154 RetIndex:1228
CompName:2,6-Octadien-1-ol, 3,7-dimethyl-, (E)- $$ trans-Geraniol $$ Guaniol $$ Lemonol $$ Nerol $$ Neryl Alcohol $$ Geraniol $$ trans-3,7-Dimethyl

Hit#:3 Entry:9547 Library:NIST08s.LIB
SI:94 Formula:C10H18O CAS:106-24-1 MolWeight:154 RetIndex:1228
CompName:2,6-Octadien-1-ol, 3,7-dimethyl-, (E)- $$ trans-Geraniol $$ Guaniol $$ Lemonol $$ Nerol $$ Neryl Alcohol $$ Geraniol $$ trans-3,7-Dimethyl

Hit#:4 Entry:9598 Library:NIST08s.LIB
SI:93 Formula:C10H18O CAS:624-15-7 MolWeight:154 RetIndex:1228
CompName:2,6-Octadien-1-ol, 3,7-dimethyl- $$ 3,7-Dimethyl-2,6-octadien-1-ol $$ (2E)-3,7-Dimethyl-2,6-octadien-1-ol # $$

Hit#:5 Entry:17360 Library:NIST08.LIB
SI:93 Formula:C10H18O CAS:624-15-7 MolWeight:154 RetIndex:1228
CompName:2,6-Octadien-1-ol, 3,7-dimethyl- $$ 3,7-Dimethyl-2,6-octadien-1-ol $$ (2E)-3,7-Dimethyl-2,6-octadien-1-ol # $$

<< Target >>
Line#:6 R.Time:20.605(Scan#:3522) MassPeaks:299 BasePeak:41.05(82385)
RawMode:Averaged 20.600-20.610(3521-3523) BG Mode:Calc. from Peak

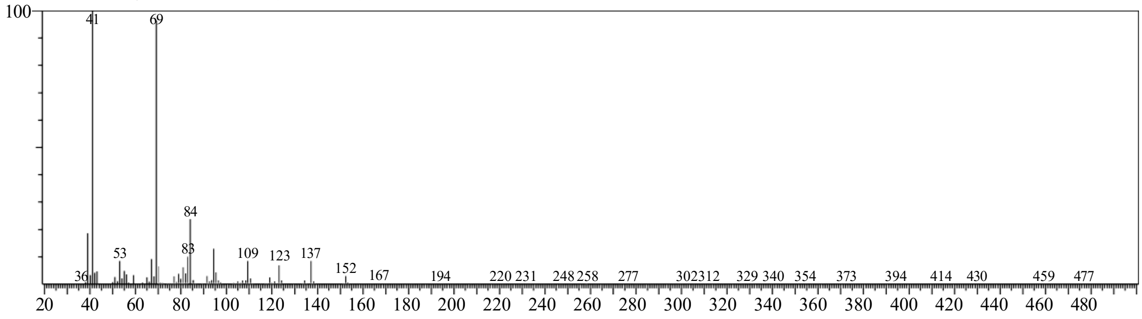

Hit#:1 Entry:9148 Library:NIST08s.LIB
SI:97 Formula:C10H16O CAS:5392-40-5 MolWeight:152 RetIndex:1174
CompName:2,6-Octadienal, 3,7-dimethyl- $$ Citral $$ 3,7-Dimethyl-2,6-octadienal $$ Citral,c&t $$ cis,trans-Citral $$ Geranial $$ NCI-C56348 $$ 3,7-Di

Hit#:2 Entry:16177 Library:NIST08.LIB
SI:97 Formula:C10H16O CAS:141-27-5 MolWeight:152 RetIndex:1174
CompName:2,6-Octadienal, 3,7-dimethyl-, (E)- $$ .alpha.-Citral $$ (E)-Citral $$ trans-Citral $$ trans-3,7-Dimethyl-2,6-octadienal $$ Citral a $$ Geranalde

Hit#:3 Entry:9151 Library:NIST08s.LIB
SI:96 Formula:C10H16O CAS:141-27-5 MolWeight:152 RetIndex:1174
CompName:2,6-Octadienal, 3,7-dimethyl-, (E)- $$ .alpha.-Citral $$ (E)-Citral $$ trans-Citral $$ trans-3,7-Dimethyl-2,6-octadienal $$ Citral a $$ Geranalde

Hit#:4 Entry:16176 Library:NIST08.LIB
SI:95 Formula:C10H16O CAS:106-26-3 MolWeight:152 RetIndex:1174
CompName:2,6-Octadienal, 3,7-dimethyl-, (Z)- $$ .beta.-Citral $$ cis-Citral $$ cis-3,7-Dimethyl-2,6-octadienal $$ Citral b $$ Neral $$ Z-Citral $$ (Z)-3,7-

Hit#:5 Entry:9119 Library:NIST08s.LIB
SI:93 Formula:C10H16O CAS:141-27-5 MolWeight:152 RetIndex:1174
CompName:2,6-Octadienal, 3,7-dimethyl-, (E)- $$ .alpha.-Citral $$ (E)-Citral $$ trans-Citral $$ trans-3,7-Dimethyl-2,6-octadienal $$ Citral a $$ Geranalde

<< Target >>
Line#:7 R.Time:24.480(Scan#:4297) MassPeaks:279
BasePeak:69.05(2185) RawMode:Averaged 24.475-24.485(4296-4298) BG Mode:Calc. from Peak 100

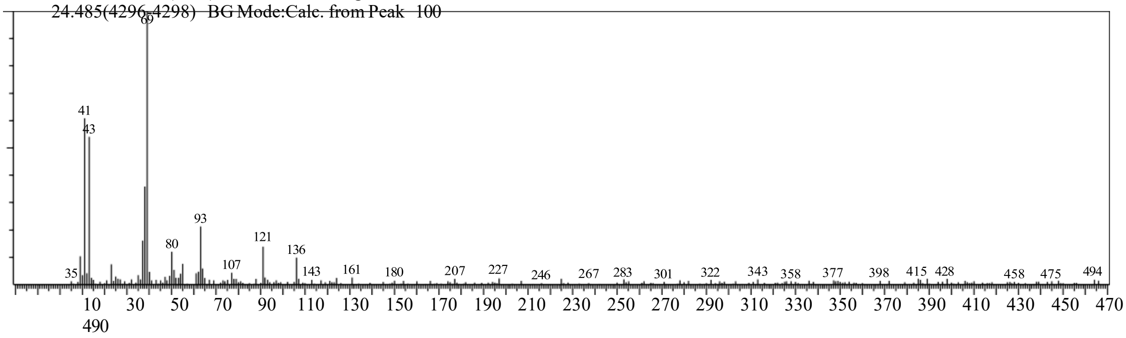

Hit#:1 Entry:16041 Library:NIST08s.LIB
SI:92 Formula:C12H20O2 CAS:105-87-3 MolWeight:196 RetIndex:1352
CompName:2,6-Octadien-1-ol, 3,7-dimethyl-, acetate, (E)- $$ Acetic acid, geraniol ester $$ Bay pine (oyster) oil $$ Geraniol acetate $$ Geranyl acetate $$ t

Hit#:2 Entry:16042 Library:NIST08s.LIB
SI:92 Formula:C12H20O2 CAS:105-87-3 MolWeight:196 RetIndex:1352
CompName:2,6-Octadien-1-ol, 3,7-dimethyl-, acetate, (E)- $$ Acetic acid, geraniol ester $$ Bay pine (oyster) oil $$ Geraniol acetate $$ Geranyl acetate $$ t

Hit#:3 Entry:16039 Library:NIST08s.LIB
SI:91 Formula:C12H20O2 CAS:141-12-8 MolWeight:196 RetIndex:1352
CompName:2,6-Octadien-1-ol, 3,7-dimethyl-, acetate, (Z)- $$ Nerol acetate $$ Neryl acetate $$ (2Z)-3,7-Dimethyl-2,6-octadienyl acetate # $$

Hit#:4 Entry:40504 Library:NIST08.LIB
SI:91 Formula:C12H20O2 CAS:105-87-3 MolWeight:196 RetIndex:1352
CompName:2,6-Octadien-1-ol, 3,7-dimethyl-, acetate, (E)- $$ Acetic acid, geraniol ester $$ Bay pine (oyster) oil $$ Geraniol acetate $$ Geranyl acetate $$ t

Hit#:5 Entry:16038 Library:NIST08s.LIB
SI:91 Formula:C12H20O2 CAS:141-12-8 MolWeight:196 RetIndex:1352
CompName:2,6-Octadien-1-ol, 3,7-dimethyl-, acetate, (Z)- $$ Nerol acetate $$ Neryl acetate $$ (2Z)-3,7-Dimethyl-2,6-octadienyl acetate # $$

<< Target >>
Line#:8 R.Time:27.845(Scan#:4970) MassPeaks:248
BasePeak:161.00(1868) RawMode:Averaged 27.840-27.850(4969-4971) BG Mode:Calc. from Peak 100

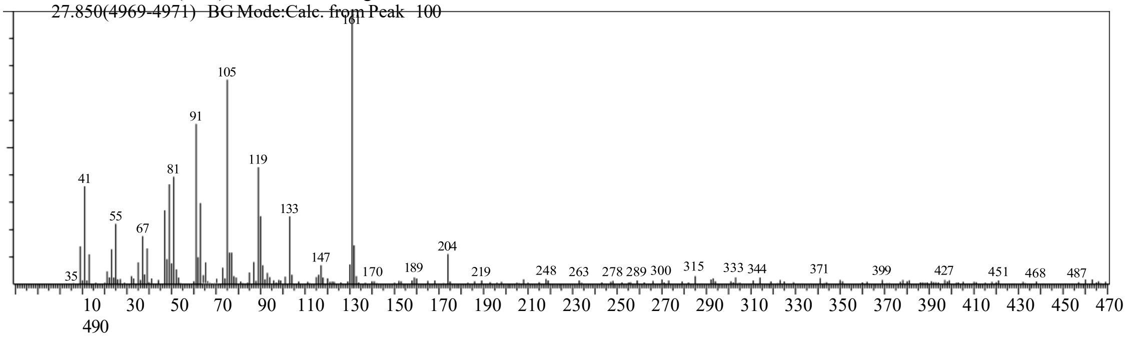

Hit#:1 Entry:45510 Library:NIST08.LIB
SI:91 Formula:C15H24 CAS:23986-74-5 MolWeight:204 RetIndex:1515
CompName:1,6-Cyclodecadiene, 1-methyl-5-methylene-8-(1-methylethyl)-, [s-(E,E)]- $$ Germacrene D $$ 8-Isopropyl-1-methyl-5-methylene-1,6-cyclode

Hit#:2 Entry:17160 Library:NIST08s.LIB
SI:89 Formula:C15H24 CAS:30021-74-0 MolWeight:204 RetIndex:1435
CompName:Naphthalene, 1,2,3,4,4a,5,6,8a-octahydro-7-methyl-4-methylene-1-(1-methylethyl)-, (1.alpha.,4a.alpha.,8a.alpha.)- $$ .gamma.-Muurolene $$ 1

Hit#:3 Entry:17158 Library:NIST08s.LIB
SI:89 Formula:C15H24 CAS:13744-15-5 MolWeight:204 RetIndex:1339
CompName:1H-Cyclopenta[1,3]cyclopropa[1,2]benzene, octahydro-7-methyl-3-methylene-4-(1-methylethyl)-, [3aS-(3a.alpha.,3b.beta.,4.beta.,7.alpha.,7aS*

Hit#:4 Entry:17163 Library:NIST08s.LIB
SI:89 Formula:C15H24 CAS:23986-74-5 MolWeight:204 RetIndex:1515
CompName:1,6-Cyclodecadiene, 1-methyl-5-methylene-8-(1-methylethyl)-, [s-(E,E)]- $$ Germacrene D $$ 8-Isopropyl-1-methyl-5-methylene-1,6-cyclode

Hit#:5 Entry:17125 Library:NIST08s.LIB
SI:87 Formula:C15H24 CAS:17699-14-8 MolWeight:204 RetIndex:1344
CompName:.alpha.-Cubebene $$ 1H-Cyclopenta[1,3]cyclopropa[1,2]benzene, 3a,3b,4,5,6,7-hexahydro-3,7-dimethyl-4-(1-methylethyl)-, [3aS-(3a.alpha.,3b.
